# Neonatal impact of maternal biomarker screening for risk of preterm birth with targeted interventions (PRIME): A multicenter, randomized, controlled trial

**DOI:** 10.1002/pmf2.70202

**Published:** 2026-01-06

**Authors:** Brian K. Iriye, John M. O'Brien, Christopher S. Ennen, Perry S. Barrilleaux, Jill A. Berkin, Anna Palatnik, Moeun Son, Cynthia Gyamfi‐Bannerman, Mollie McDonnold, Glenn R. Markenson, Anthony C. Sciscione, Joseph R. Biggio, Samuel Wolf, Scott A. Sullivan, Michael Walker, Babak Shahbaba, Jannik Godt, Stephen P. Pound

**Affiliations:** ^1^ High Risk Pregnancy Center Hera Women's Health Las Vegas Nevada USA; ^2^ Department of Obstetrics and Gynecology University of Kentucky Health Care Lexington Kentucky USA; ^3^ Department of Obstetrics and Gynecology, University of Virginia School of Medicine Charlottesville Virginia USA; ^4^ Department of Obstetrics and Gynecology Louisiana State University Health Science Center Shreveport Louisiana USA; ^5^ Department of Obstetrics, Gynecology, and Reproductive Science Icahn School of Medicine at Mount Sinai New York New York USA; ^6^ Department of Obstetrics and Gynecology Medical College of Wisconsin Milwaukee Wisconsin USA; ^7^ Department of Obstetrics, Gynecology, and Reproductive Science Yale University New Haven Connecticut USA; ^8^ Department of Obstetrics, Gynecology, and Reproductive Sciences University of California San Diego La Jolla California USA; ^9^ Austin Maternal‐Fetal Medicine Austin Texas USA; ^10^ Department of Obstetrics and Gynecology Boston University Chobanian and Avedisian School of Medicine Boston Massachusetts USA; ^11^ Department of Obstetrics and Gynecology ChristianaCare Health System Newark Delaware USA; ^12^ Department of Maternal‐Fetal Medicine Delaware Center for Maternal Fetal Medicine Newark Delaware USA; ^13^ Department of Obstetrics and Gynecology, Ochsner Health New Orleans Louisiana USA; ^14^ Emerald Coast OBGYN Clinical Research Panama City Florida USA; ^15^ Department of Obstetrics and Gynecology, Inova Health Fairfax Virginia USA; ^16^ Walker Bioscience Carlsbad California USA; ^17^ Department of Statistics, University of California, Irvine Irvine California USA; ^18^ JGConsult Nærum Denmark; ^19^ Virginia Physicians for Women North Chesterfield Virginia USA; ^20^ Present address: Department of Obstetrics and Gynecology Weill Cornell Medical College New York NY USA

**Keywords:** biomarker screening, care management, low‐dose aspirin, neonatal intensive care unit (NICU), neonatal morbidity, preterm birth, vaginal progesterone

## Abstract

**Background:**

Preterm birth remains a leading cause of neonatal morbidity and mortality, yet clinicians possess limited tools to identify at‐risk pregnancies and implement effective interventions. This study evaluated whether knowledge of a mid‐second‐trimester maternal blood biomarker test result, combined with an intervention bundle for those identified as being at higher risk, improves neonatal outcomes in a population at low overall risk for spontaneous preterm birth.

**Methods:**

PRIME was a multicenter, randomized, controlled trial at 19 US sites. Participants with singleton pregnancies at 18–20^6/7^ weeks’ gestation underwent biomarker testing with the previously validated insulin‐like growth factor‐binding protein 4 to sex hormone‐binding globulin (IGFBP4/SHBG) ratio and were randomized to an open‐label screen‐guided care arm or a blinded routine care arm. Higher‐risk participants in the screen‐guided care arm received a bundled intervention consisting of daily vaginal progesterone, low‐dose aspirin, and weekly telephonic nurse support. The co‐primary outcomes, analyzed in an intent‐to‐treat population, were: (1) a composite index of neonatal morbidity and mortality and (2) neonatal hospital length of stay. Additional outcomes were gestational age at birth, neonatal intensive care unit (NICU) admissions, and NICU length of stay. Outcomes were adjusted for parity, pre‐enrollment aspirin use, and COVID positivity. Recruitment was halted for efficacy at the interim analysis, and all enrolled participants were followed to delivery and neonatal outcome assessment.

**Results:**

The enrolled population included 5018 participants with delivery data available for 4806, comprising the intent‐to‐treat population (2416 screen‐guided care, 2390 routine care). Screen‐guided care was associated with reduced composite neonatal morbidity (odds ratio [OR], 0.80; 95% confidence interval [CI], 0.66–0.96; *p* = 0.015), shortened neonatal hospital stays (incidence rate ratio [IRR], 0.95; 95% CI, 0.92–0.98; *p* = 0.004), and fewer NICU admissions.

**Conclusion:**

In low‐risk pregnancies, care guided by a maternal IGFBP4/SHBG blood test with targeted interventions improved neonatal outcomes. These findings suggest a potential role for IGFBP4/SHBG screen‐guided care in enhancing early risk detection and informing strategies to reduce events related to preterm birth.

**Trial Registration:**

ClinicalTrials.gov identifier: NCT04301518

## 1 INTRODUCTION

Preterm birth results from multiple diverse etiologies, complicating risk assessment, prevention, and treatment. In 2020, an estimated 13.4 million preterm births contributed to approximately 900,000 infant deaths worldwide [1]. The US preterm birth rate was 10.4% in 2024 [2] and has remained essentially unchanged for decades, contributing to significant neonatal morbidity and mortality [3]. The annual economic burden of preterm birth in the United States reflects its substantial impact on healthcare systems, with additional, far‐reaching consequences for families, employers, and society at large. These costs encompass prolonged neonatal intensive care, long‐term medical and developmental support, lost parental productivity, and increased educational and social service needs for children born preterm [4].

Identifying women at risk for preterm birth remains a significant challenge, as current strategies often overlook large segments of the population. Risk assessment tools that rely primarily on clinical history and demographic factors fail to detect the majority of at‐risk pregnancies, limiting the effectiveness of prevention efforts [5, 6]. Notably, up to half of preterm births occur in the absence of identifiable risk factors, limiting the effectiveness of currently available screening strategies [7].

The search for reliable preterm birth screening methods has driven efforts to find informative biomarkers. A mid‐second trimester blood test measuring the ratio of circulating maternal insulin‐like growth factor‐binding protein 4 to sex hormone‐binding globulin (IGFBP4/SHBG) has emerged as a promising predictor of spontaneous preterm birth. Validated in two clinical studies, this test demonstrated a sensitivity of 75% and a specificity of 74%, with an area under the receiver operating characteristic curve of 0.75 [8, 9]. A test score indicating a ≥15% risk of spontaneous preterm birth—roughly twice the US average for singleton pregnancies—highlights a population at elevated risk, in whom studies have shown extended neonatal hospital stays and increased neonatal morbidity [8, 9]. In the placenta, pregnancy‐associated plasma protein‐A (PAPP‐A) cleaves IGFBP4, increasing local insulin‐like growth factor (IGF) bioavailability [10]. This process has a role in regulation of trophoblast invasion, vascular development, and placental growth, functions essential for sustaining pregnancy [11, 12]. Elevated IGFBP4 limits free IGF, restricting these functions and contributing to impaired placentation. Such alterations have been linked with fetal growth restriction and adverse outcomes including preterm birth [13, 14]. SHBG is expressed by the placenta [15] and is downregulated by inflammatory cytokines [16]. Reduced SHBG may lead to increased effects of local androgen‐ and estrogen‐mediated pathways, providing a plausible link between inflammation at the maternal‐fetal interface and the decreased placental SHBG expression noted in spontaneous preterm birth [8, 17] Given the plausible mechanisms related to inflammation and placental function reflected by these biomarkers, we propose that an elevated IGFBP4/SHBG ratio may contribute to identifying pregnancies at risk for spontaneous preterm birth who may benefit from targeted interventions aimed at these pathways. Currently, evidence‐based preterm birth interventions, when used individually, have limited benefit. While low‐dose aspirin is commonly prescribed during pregnancy, its preventive effect on adverse outcomes of prematurity appears modest [18, 19]. In individuals without a history of preterm birth, vaginal progesterone has been shown to be effective for individuals with a short cervix, but this diagnosis is relatively infrequent in the overall pregnant population [20]. Care management services, typically involving telephonic nurse support, have demonstrated improved outcomes in Medicaid‐insured populations; however, their implementation remains limited and inconsistent [21, 22].

Recognizing the complex and multifactorial nature of preterm birth, we hypothesized that an IGFBP4/SHBG screen‐guided, comprehensive care bundle combining pharmacologic therapy with structured care management would more effectively address the diverse pathways leading to preterm birth and meaningfully improve neonatal outcomes.

## 2 METHODS

### 2.1 Study design

The Prematurity Risk Assessment Combined with Clinical Interventions for Improving Neonatal OutcoMEs (PRIME) trial (NCT04301518) was a multicenter, randomized, controlled trial conducted at 19 US centers to evaluate whether knowing the results of a maternal IGFBP4/SHBG blood test for risk of preterm birth could improve neonatal outcomes relative to routine care. Participants were randomized into two parallel groups: the screen‐guided care arm, in which test results were disclosed, and the routine care arm, in which test results were withheld. Within the screen‐guided care arm, women identified as higher risk for preterm birth were offered a bundled intervention.

### 2.2 Participants

Participants were enrolled from November 6, 2020 to December 6, 2023 and included pregnant individuals ≥18 years of age with singleton gestations between 18^0/7^ and 20^6/7^ weeks’ gestation, confirmed by standard dating criteria [23]. Inclusion criteria required informed consent, maternal and fetal anatomic ultrasound completion after 18^0/7^ weeks’ gestation, including standard cervical length assessment, a singleton gestation, and expected neonatal data accessibility. Exclusion criteria included a history of spontaneous preterm birth, cervical length < 25 mm, major fetal anomalies, use of progesterone after 13^6/7^ weeks, and other conditions associated with high risk for preterm delivery (e.g., placenta previa, uterine anomalies, severe COVID‐19, or chronic maternal disease requiring intensive surveillance). Full inclusion and exclusion criteria are in Table S1. Initial written informed consent, available in English or Spanish, addressed study enrollment and blood collection, with disclosure that the bundled interventions might be offered depending on test results. For participants in the screen‐guided care arm who screened at higher risk, a second consent process was undertaken prior to initiation of the intervention bundle. This process included a detailed description of the intervention strategy. Each consent process offered optional video materials summarizing the study and protocol. Participants who declined the intervention remained enrolled in the screen‐guided care arm.

### 2.3 Randomization and masking

After sample receipt by the laboratory and before IGFBP4/SHBG testing, participants were randomized 1:1 to either the screen‐guided care arm or the routine care arm using computer‐generated simple randomization managed by an independent data coordinating center (Carelon Research, Wilmington, DE, USA). The study was open‐label regarding study arm assignment, and test results were disclosed to participants only in the screen‐guided care arm. In the routine care arm, test results were blinded to participants, study personnel, and personnel involved in neonatal care, data collection, and outcome assessment.

### 2.4 Procedures

All participants underwent mid‐second‐trimester blood sampling for assessment of the IGFBP4/SHBG ratio via liquid chromatography‐mass spectrometry, as described previously [24]. Participants randomized to the screen‐guided care arm were classified as “higher risk” if their IGFBP4/SHBG risk score was ≥15% for preterm birth, corresponding to a threshold approximately twice the rate of spontaneous singleton preterm birth [9]. Participants in the screen‐guided care arm who tested higher risk and provided consent received a bundled intervention consisting of daily self‐administered vaginal micronized progesterone (200 mg), daily oral low‐dose aspirin (81 mg), and weekly standardized telephonic nurse care management (Table S2).

Interventions were designed to begin before 24^0/7^ weeks’ gestation and continue until 36^6/7^ weeks’ gestation or delivery. Higher‐risk participants in the screen‐guided care arm were also offered two additional transvaginal cervical length assessments, at 21^0/7^ through 23^6/7^ weeks’ gestation and 26^0/7^ through 28^6/7^ weeks’ gestation. If cervical length was ≤ 10 mm before 24^0/7^ weeks, cerclage was to be recommended for the participant, with the final determination of cerclage placement made by the physician and participant on an individualized basis. The remaining participants in the screen‐guided care arm were classified as “not higher risk” and continued to receive usual obstetric care. Routine care arm participants also received usual obstetric care.

### 2.5 Analysis populations

Analyses were performed in two predefined populations.

The intent‐to‐treat (ITT) population comprised all randomized participants with outcome data and was used for the primary analysis of all trial outcomes.

The modified intent‐to‐treat (mITT) population was prespecified as the primary analytic set in the trial statistical analysis plan (SAP). In contrast with the ITT population, the mITT population excluded participants in the screen‐guided care arm who tested as higher risk and did not consent to or initiate the intervention before 24 weeks’ gestation, as well as those diagnosed with COVID‐19 post‐randomization and evaluated in a hospital, due to the elevated risk of adverse outcomes [25]. This approach aimed to assess treatment efficacy while accounting for anticipated withdrawals, particularly related to medications of uncertain benefit or requiring undesired vaginal administration. Participants in the mITT analysis were not excluded for protocol deviations or nonadherence. The mITT population was used for interim and final efficacy analyses, consistent with the SAP.

Report of the ITT population as the primary focus of results aligns with recommended reporting standards for randomized trials and was performed in accordance with editorial guidance. Consistent with the SAP, the prespecified mITT analyses were included to support the interpretation of trial efficacy.

### 2.6 Outcomes

The study assessed two co‐primary outcomes: (1) neonatal morbidity and mortality, quantified using a composite neonatal morbidity index (NMI) adapted from Hassan et al. [20] (Table 1) and (2) neonatal hospital length of stay. Neonatal hospital length of stay was selected as a co‐primary outcome over neonatal intensive care unit (NICU) length of stay because it reflects the entire neonatal care episode, including the duration of NICU stay. By contrast, NICU admission and discharge practices, particularly among late preterm and early term infants, exhibit substantial inter‐institutional variation and are frequently influenced by local protocols and bed availability rather than uniform indicators of illness severity [26, 27, 28].

**TABLE 1:** Neonatal composite morbidity/mortality index scoring.

Composite neonatal morbidity index scale^a^	
0‐to‐4 scale integrating neonatal intensive care unit (NICU) length of stay	0 = No events 1 = One event for (RDS, BPD, IVH Grade III or IV, any PVL, proven sepsis, or NEC) or 1–4 days in the NICU, and no neonatal mortality 2 = Two events or from 5 to 20 days in the NICU, and no neonatal mortality 3 = Three or more events or >20 days in the NICU, and no neonatal mortality 4 = Neonatal mortality
**Definitions of neonatal morbidity events included in scale**	
**Category**	**Description**
Intraventricular hemorrhage (IVH)	Determined by cranial ultrasound or computed tomography ‐ Grade III: intraventricular hemorrhage with ventricular dilatation ‐ Grade IV: intraventricular hemorrhage with ventricular dilatation and parenchymal extension
Periventricular leukomalacia (PVL)	Determined by cranial ultrasound ‐ Any PVL ‐ Cystic PVL
Necrotizing enterocolitis (NEC)	Stage III: Surgical—Advanced (treatment was surgical)
Respiratory distress syndrome (RDS) or hyaline membrane disease (HMD)	Requires both diagnosis and oxygen therapy.Must include: ‐ Oxygen therapy (fraction of inspired oxygen (FiO_2_) ≥ 0.40) until infant death or ≥24 h or continuous positive airway pressure (CPAP) and ‐ Clinical diagnosis of RDS or HMD
Bronchopulmonary dysplasia (BPD)	Treatment with >21% oxygen for at least 28 days, or Oxygen dependence after 36 weeks post‐conceptional age
Sepsis	Must include:Blood culture‐proven sepsis, andA clinically ill infant with infection defined as: ‐ Bacterial sepsis of the newborn ‐ Streptococcal sepsis ‐ Severe sepsis
Neonatal mortality	Death within 28 days of delivery

^a^ Adapted from Hassan et al. [20].

Secondary outcomes were (1) NICU length of stay and (2) gestational age at birth.

Additional outcomes included NICU admission, categorical gestational age at birth, and birthweight.

Adverse events were collected as reported by study centers up to 14 days postdelivery and evaluated by the principal investigator regarding relatedness, severity, and seriousness. Investigators were required to report serious adverse events within 24 hours of becoming aware of the event for review by the independent Medical Monitor. All reported adverse events were evaluated by the independent Medical Monitor before presentation to the independent Data and Safety Monitoring Board (DSMB).

The outcomes examined in this study comprise all but one (late neurodevelopmental morbidity) of the 13 Global Obstetrics Network core outcomes for evaluating preterm birth prevention approaches [29].

### 2.7 Power and sample size analysis

Power and sample size estimation attempted to account for the baseline risk of adverse neonatal outcomes related to prematurity, the IGFBP4/SHBG test screen‐positive rate, compliance rates, the efficacy of the intervention bundle, and missing data within the ITT population. As detailed in the Supporting Information, simulations used a screen‐positive rate estimated from prior IGFBP4/SHBG biomarker studies [30, 31] and effect sizes estimated from published trials on progesterone and care management [21, 32, 33, 34, 35, 36]. Based on these simulations and a projected loss to follow‐up of 16%, the target sample size was 6500 participants.

### 2.8 Statistical analysis

Final trial outcomes were analyzed in the ITT population. Analysis of co‐primary outcomes used ordinal logistic regression for neonatal morbidity and mortality and Poisson regression for neonatal hospital length of stay. Neonatal hospital length of stay was evaluated using Poisson regression to account for the highly skewed distribution, dominated by short hospital stays for most neonates.

Co‐secondary outcomes were analyzed using a zero‐inflated Poisson model for NICU length of stay and Poisson regression for gestational age at birth. The zero‐inflated Poisson model was used for NICU length of stay to account for the high number of patients with zero NICU days due to lack of NICU admission.

NICU admissions, a binary variable, was assessed using logistic regression [37].

Outcomes were analyzed both unadjusted and adjusted for prespecified covariates: parity, pre‐enrollment low‐dose aspirin use, and COVID‐19 positivity. COVID‐19 status was included given its uncertain impact on preterm birth at the study's initiation early in the pandemic. Using covariate adjustment in randomized controlled trials increases statistical power, thereby achieving the required precision with fewer participants. This enhances trial efficiency, reduces participant burden, and aligns with ethical principles that require the number of participants to be kept to the minimum necessary through efficient statistical methods [38, 39]. *p* values were not adjusted for multiple testing in secondary, exploratory, or sensitivity analyses. Trial outcomes were further examined in the ITT population using multiple imputation for missing data.

The trial was designed with a prespecified interim analysis of screen‐guided care efficacy in the mITT population when approximately 1400 eligible participants per arm had delivered with outcome data. For this analysis, boundary conditions were calculated using NCSS PASS 2020 [40]. The O'Brien‐Fleming [41] nominal *p* values for stopping trial enrollment were 0.0056 for the interim and 0.0482 for the final analyses. Type I error in testing the two co‐primary endpoints was controlled by Holm's method [42], requiring that the co‐primary outcome with the lowest *p* value reach <0.0028 to be significant at the interim and <0.0241 at the final analysis. The second co‐primary outcome was then tested at the full alpha of 0.0056 at the interim and 0.0482 at the final analysis. The DSMB recommended halting trial enrollment for efficacy after the interim analysis results met the prespecified stopping criterion of a significant reduction in one co‐primary outcomes (neonatal length of hospital stay, hazard ratio [HR], 0.49; 95% confidence interval [CI], 0.39–0.60; *p *< 0.001).

Interim analysis methodology and results, along with details on power analysis and multiple imputation, are in the Supporting Information.

## 3 RESULTS

### 3.1 Participant disposition

**FIGURE 1:**
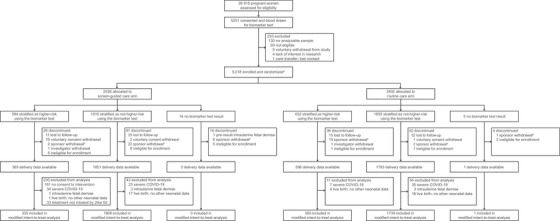
CONSORT diagram. ^a^IGFBP4/SHBG analysis occurred after randomization. ^b^Sponsor withdrawals (*n* = 54/5018, 1.1%) occurred prior to and without knowledge of maternal or neonatal outcomes and resulted from: (1) Delayed laboratory test result reporting, preventing inclusion before the intervention start deadline (*n* = 11). (2) An initial laboratory processing error that delayed intervention bundle implementation beyond the start deadline (*n* = 41). (3) Unblinding of a single routine care arm participant due to a reported result (*n* = 1). (4) Reporting of a not‐higher‐risk result with percentage risk versus masked binary risk (*n* = 1). Neonatal and delivery outcomes were not assessed for these participants.

Between November 6, 2020, and December 6, 2023, 26,915 pregnant individuals were screened, and 5018 were enrolled and randomized to either the screen‐guided care arm (*n* = 2526) or the routine care arm (*n* = 2492) (Figure 1). Participants were enrolled during pregnancy, and follow‐up continued through maternal and neonatal hospital discharge.

All participants underwent IGFBP4/SHBG testing. In the screen‐guided care arm, 23.5% (594/2526) were identified as higher‐risk and offered the bundled intervention. Of these individuals, 57.7% (343/594) accepted and initiated the interventions before 24 weeks’ gestation. In the routine care arm, 25.4% (632/2492) were identified as higher‐risk and received usual care. Participants randomized to study arms were similar across study arms with respect to baseline maternal characteristics (Table 2).

**TABLE 2:** Maternal baseline characteristics.

Characteristic	Screen‐guided care (*n* = 2526)	Routine care (*n* = 2492)	*p* value (*n* = 5018)
Maternal age—mean (SD) (yr)	31 (6.0)	30 (6.0)	0.148
Unknown	0	0	
Race—no. (%)^b^			0.159
American Indian/Alaskan Native	8 (0.3%)	8 (0.3%)	
Asian	193 (7.6%)	171 (6.9%)	
Black/African American	351 (13.9%)	375 (15.0%)	
Native Hawaiian/Pacific Islander	16 (0.6%)	13 (0.5%)	
White	1616 (64.0%)	1624 (65.2%)	
Multiracial	27 (1.1%)	39 (1.6%)	
Declined to answer	315 (12.5%)	262 (10.5%)	
Ethnic group—no. (%)^a^			0.866
Hispanic/Latina	454 (18.0%)	436 (17.5%)	
Non‐Hispanic/Non‐Latina	2002 (79.3%)	1990 (79.9%)	
Declined to answer	70 (2.8%)	66 (2.6%)	
Insurance type—no. (%)			0.285
Medicaid	623 (24.7%)	659 (26.4%)	
Commercial	1845 (73.0%)	1771 (71.1%)	
Self‐pay	57 (2.3%)	62 (2.5%)	
Missing	1 (0.0%)	0 (0.0%)	
Gravidity—mean (SD)	2.0 (2.0)	2.0 (2.0)	0.480
Parity—no. (%)			0.788
Nulliparous	1223 (48.4%)	1216 (48.8%)	
Multiparous	1303 (51.6%)	1276 (51.2%)	
Prepregnancy BMI^b^—mean (SD)	28.0 (7.3)	27.6 (7.0)	0.084
Prepregnancy BMI categories—no. (%)			
Underweight (<18.5)	64 (2.5%)	72 (2.9%)	
Normal weight (18.5–24.9)	978 (38.8%)	1016 (40.8%)	
Overweight (25–29.9)	665 (26.4%)	643 (25.8%)	
Obesity class I (30–34.9)	406 (16.1%)	382 (15.3%)	
Obesity class II (35–39.9)	225 (8.9%)	227 (9.1%)	
Obesity III (≥40)	182 (7.2%)	149 (6.0%)	
Preexisting diabetes (Type II)—no. (%)			0.160
Yes	78 (3.1%)	95 (3.8%)	
No	2448 (96.9%)	2397 (96.2%)	
Chronic hypertension—no. (%)			0.743
Yes	169 (6.7%)	161 (6.5%)	
No	2357 (93.3%)	2331 (93.5%)	
Pre‐enrollment aspirin use (81 mg)—no. (%)			0.815
Yes	694 (27.5%)	692 (27.8%)	
No	1832 (72.5%)	1800 (72.2%)	

*Note*: Other than insurance type, there were no missing values. Descriptive statistics reported. Instances where percentages do not total 100% are due to rounding.

Abbreviations: BMI, body mass index; SD, standard deviation.

^a^ Race and ethnic group were self‐reported.

^b^ BMI is the weight (kg) divided by the square of the height (m).

The ITT population comprised 4806 participants with outcome data (2416 screen‐guided care, 2390 routine care), representing 95.8% of all randomized participants.

### 3.2 Outcomes

**FIGURE 2:**
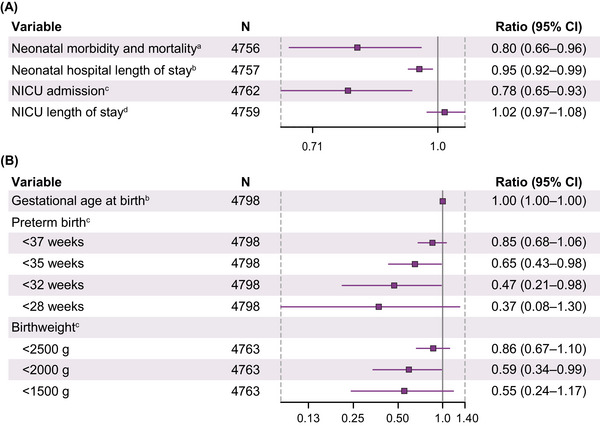
Adjusted outcomes in the screen‐guided care arm versus the routine care arm in the intent‐to‐treat population. 95% CI, 95% confidence interval; NICU, neonatal intensive care unit. Data were adjusted for covariates parity, pre‐enrollment aspirin use, and COVID‐19 positivity. The intent‐to‐treat population was used in all analyses. Lower ratios denote lower rates of these events in the screen‐guided care arm versus in the routine care arm. ^a^Odds ratio; ordinal logistic regression analysis. ^b^Incidence rate ratio; Poisson regression analysis. ^c^Odds ratio; logistic regression analysis. ^d^Incidence rate ratio; zero‐inflated Poisson regression analysis.

Composite neonatal morbidity and mortality in the screen‐guided care arm decreased by 20% compared to routine care (odds ratio [OR], 0.80; 95% CI, 0.66–0.96; *p *= 0.015). Neonatal hospital length of stay was also reduced in the screen‐guided care arm (incidence rate ratio [IRR], 0.95; 95% CI, 0.92–0.98; *p *< 0.004). Outcomes are summarized in Table 3 and Figure 2.

**TABLE 3:** Primary, secondary, and exploratory outcomes in the intent‐to‐treat population.

Outcome	*N* ^a^	Screen‐guided care	Routine care	Unadjusted (95% CI)	*p* value	Adjusted (95% CI)^b^	*p* value
**Co‐primary outcomes**							
Neonatal morbidity and mortality^c^	4756			0.80 (0.67–0.96)	0.016	0.80 (0.66–0.96)	0.015
Neonatal hospital length of stay [mean (SD)]	4757	2.92 (6.74)	3.07 (7.95)	0.95 (0.92–0.98)^d^	0.003	0.95 (0.92–0.98)^d^	0.004
**Secondary and exploratory outcomes**							
NICU admission [*n*/*N* (%)]	4762	245/2392 (10.2)	303/2370 (12.8)	0.78 (0.65–0.93)^e^	0.006	0.78 (0.65–0.93)^e^	0.006
NICU length of stay [mean days (SD)]	4759	1.10 (6.93)	1.34 (8.12)	1.01 (0.96–1.06)^f^	0.682	1.02 (0.97–1.08)^f^	0.395
Gestational age at birth [mean weeks (SD)]	4798	38.87 (1.48)	38.86 (1.70)	1.00 (1.00–1.00)^d^	0.80	1.00 (1.00–1.00)^d^	0.80

Abbreviations: CI, confidence interval; SD, standard deviation.

^a^ Total of participants with outcome data, excluding missing data and intrauterine fetal demise.

^b^ Adjusted for parity, pre‐enrollment aspirin use, and COVID‐19 positivity.

^c^ Data are based on categorical neonatal morbidity index scores. For counts and percentages, see Table 5. Odds ratio; ordinal logistic regression.

^d^ Incidence rate ratio; Poisson regression analysis.

^e^ Logistic regression analysis.

^f^ Incidence rate ratio; zero‐inflated Poisson count regression analysis.

For neonates admitted to the NICU, the length of stay did not differ significantly between study arms. However, the likelihood of NICU admission was significantly decreased in the screen‐guided care arm (OR, 0.78; 95% CI, 0.65–0.93; *p* = 0.006). There were 58 fewer NICU admissions in the screen‐guided care arm, equating to a number needed to screen (NNS) of 38.5 participants (95% CI, 24.09–118.05; *p* = 0.016) to prevent one NICU admission (Table 4). This translated to an overall reduction of 545 NICU days in the screen‐guided care arm. These outcomes correspond to an NNS of 4.2 (95% CI, 3.03–6.70; *p* = 0.014) to prevent a single NICU day. Multiple imputation, employed to account for the 4.3% of participants with missing data, supported the primary and secondary outcome conclusions (Table S3).

**TABLE 4:** Neonatal intensive care unit admission rate and days in the intent‐to‐treat population.

	Screen‐guided care (*n* = 2416)	Routine care (*n* = 2390)	*p* value	Number needed to screen (95% CI)
NICU admission [*n*, (%)]	245 (10.2%)	303 (12.8%)	0.006^a^	38.5 (24.09–118.05)
NICU length of stay ^a^ [total days, (mean)]	2630 (1.10)	3175 (1.34)	0.014^b^	4.2 (3.03–6.70)

*Note*: NNS calculated from the mean difference in NICU days or the absolute risk reduction of NICU admission between arms using the designated ITT population. 95% CIs for NNS were calculated using logistic regression and Poisson regression.

Abbreviations: 95% CI, 95% confidence interval; ITT, intent‐to‐treat; NICU, neonatal intensive care unit; NNS, number needed to screen.

^a^ Pearson's chi‐squared test.

^b^ Wilcoxon rank sum test.

The mean gestational age at birth did not differ between groups. Given that singleton preterm births occur in a small minority of cases, overall changes in mean gestational age were unlikely. However, there were fewer preterm births before 32 and 35 weeks’ gestation in the screen‐guided care arm. The directionality of the treatment effect also became more pronounced at earlier gestational ages, as shown in Figure 2 forest plots. A similar pattern was observed for birthweight, with greater effects at lower birthweights and a reduction in births under 2000 grams.

Additional neonatal and maternal outcomes are summarized in Table 5. In the screen‐guided care arm, there were 19 fewer index morbidities (67 vs. 86) and 16 fewer neonates affected by an index morbidity (56 vs. 72) compared with the routine care arm. Per the study protocol, all patients in the higher‐risk subgroup of the screen‐guided care arm were to undergo two follow‐up transvaginal cervical length measurements. Among those assessed, the shortest cervical length was 14 mm at 27^5/7^ weeks’ gestation. No participants met the threshold (<10 mm) for cerclage placement at less than 24 weeks’ gestation. Sixteen participants were identified with a short cervix (≤25 mm) after study inclusion on protocol‐driven follow‐up transvaginal sonography. Of these, one experienced spontaneous preterm birth at 35^6/7^ weeks, and one had a medically indicated preterm delivery at 34^0/7^ weeks for oligohydramnios with fetal growth restriction. The remaining 14 participants delivered at term.

**TABLE 5:** Neonatal and maternal outcomes in the intent‐to‐treat population.^a^

Outcome	Screen‐guided care	Routine care	*p* value
Neonatal	(*n* = 2416)	(*n* = 2390)	(*n* = 4806)
Birth weight—mean (SD) (g)	3312 (511)	3300 (530)	0.724
<2500 g	120 (5.0%)	137 (5.8%)	0.234
<2000 g	22 (0.9%)	37 (1.6%)	0.044
<1500 g	10 (0.4%)	18 (0.8%)	0.121
Missing	15	20	
Neonatal morbidity index score—no. (%)			0.079
0	2151 (90.1%)	2080 (87.8%)	
1	112 (4.7%)	143 (6.0%)	
2	95 (4.0%)	111 (4.7%)	
3	30 (1.3%)	32 (1.4%)	
4	0 (0%)	2 (0.1%)	
Missing	23	19	
Neonatal morbidity index score severity level—no. (%)			
≥1	237 (9.9%)	288 (12.2%)	0.014
≥2	125 (5.2%)	145 (6.1%)	0.185
≥3	30 (1.3%)	34 (1.4%)	0.591
Missing	23	19	
Neonatal morbidities included in index score—no. (%)			
Intraventricular hemorrhage grade III/IV	1 (0.0%)	3 (0.1%)	0.345
Periventricular leukomalacia	0 (0%)	4 (0.2%)	0.061
Necrotizing enterocolitis	0 (0%)	1 (0.0%)	0.498
Respiratory distress syndrome	55 (2.3%)	64 (2.7%)	0.373
Bronchopulmonary dysplasia	7 (0.3%)	5 (0.2%)	0.573
Blood culture‐proven sepsis	4 (0.2%)	7 (0.3%)	0.358
Neonatal mortality	0 (0%)	2 (0.1%)	0.247
Missing	23	19	
Total neonatal morbidities/mortality contributing to index score—no.	67	86	0.105
Total neonates with any index morbidity—no. (%)	56 (2.3%)	72 (3.0%)	0.138
Morbidities per neonate—no. (%)			
0	2332 (97.7%)	2296 (97.0%)	0.093
1	48 (2.0%)	61 (2.6%)	
2	5 (0.2%)	10 (0.4%)	
3	3 (0.1%)	0 (0%)	
4	0 (0%)	0 (0%)	
5	0 (0%)	1 (0.0%)	
Missing	23	19	
Neonatal length of hospital stay among all neonates (days)			
Mean (SD)	2.92 (6.74)	3.07 (7.95)	0.064
Median (range)	0 (0–160)	0 (0–188)	
Missing	21	20	
NICU admissions—no. (%)	245 (10.2%)	303 (12.8%)	0.006
In deliveries <37 weeks gestation	84 (3.5%)	106 (4.5%)	0.262
In deliveries ≥37 weeks gestation	161 (6.7%)	197 (8.3%)	0.030
Missing	19	17	
NICU length of stay among all neonates (days)			
Mean (SD)	1.10 (6.93)	1.34 (8.12)	0.014
Median (range)	0 (0‐160)	0 (0‐188)	
Missing	21	18	
NICU length of stay in morbidity index score categories—no.			0.389
< 1	2187 (91.5%)	2134 (90.1%)	
1‐4	79 (3.3%)	90 (3.8%)	
5‐20	94 (3.9%)	111 (4.7%)	
> 20	30 (1.3%)	34 (1.4%)	
Missing (NICU length of stay)	21	18	
Gestational age at birth (week)			
Mean (SD)	38.87 (1.48)	38.86 (1.70)	0.278
Preterm gestational age at birth (week)—no. (%)			
<37	159 (6.6%)	184 (7.7%)	0.134
<35	40 (1.7%)	60 (2.5%)	0.038
<32	10 (0.4%)	21 (0.9%)	0.044
<28	3 (0.1%)	8 (0.3%)	0.127
Missing	0	0	

**Maternal**	**(*n* = 2526)**	**(*n* = 2492)**	**(*n* = 5018)**
Cerclage performed—no. (%)	0 (0.0%)	0 (0.0%)	
Tocolytics administered—no. (%)			
Yes	211 (8.8%)	223 (9.4%)	0.478
Missing	118	110	
Antenatal corticosteroids administered—no. (%)			
Yes	106 (4.4%)	139 (5.8%)	0.024
Missing	115	111	
Delivery type—no. (%)			0.257
Spontaneous vaginal	1515 (62.8%)	1526 (64.0%)	
Assisted vaginal	89 (3.7%)	85 (3.6%)	
Lower segment cesarean section, no labor	386 (16.0%)	352 (14.8%)	
Lower segment cesarean section, in labor	399 (16.5%)	388 (16.3%)	
Classical cesarean section, no labor	9 (0.4%)	21 (0.9%)	
Classical cesarean section, in labor	12 (0.5%)	11 (0.5%)	
Dilation and evacuation	0 (0%)	0 (0%)	
Missing	109	106	
Primary diagnosis for preterm hospital admission at time of delivery–no.^b^			0.242
Spontaneous preterm labor	38	51	
Preterm premature rupture of membranes	31	38	
Preeclampsia/gestational hypertension	60	53	
Bleeding and/or abruption	4	12	
Intrauterine fetal demise	5	3	
Non‐reassuring fetal status and/or severe fetal growth restriction	13	15	
Other maternal indications	9	19	
Oligohydramnios	7	4	
Other	3	2	
Primary diagnosis for term hospital admission at time of delivery—no.^b^			0.451
Preeclampsia/gestational hypertension	152	113	
Fetal growth restriction	47	32	
Placental abruption	8	8	
Gestational diabetes	43	42	
Oligohydramnios	19	23	
Additional diagnoses^b^			
Fetal growth restriction	128 (5.3%)	90 (3.8%)	0.011
Febrile illness	26 (1.1%)	24 (1.0%)	0.807
HELLP syndrome	4 (0.2%)	7 (0.3%)	0.356
Oligohydramnios	63 (2.6%)	66 (2.8%)	0.741
Polyhydramnios	89 (3.7%)	80 (3.4%)	0.527
Placenta previa	32 (1.3%)	29 (1.2%)	0.730
Velamentous cord insertion	15 (0.6%)	9 (0.4%)	0.230
Vaginal bleeding ≥19 weeks gestation	86 (3.6%)	68 (2.9%)	0.161
Vasa previa	4 (0.1%)	1 (0.0%)	0.625

*Note*: Intrauterine fetal demise (*n* = 8) is not accounted for in calculations of neonatal outcomes. Percentages exclude missing values where reported. *p* values are not adjusted for multiple comparisons. Wilcoxon rank sum test is used for continuous variables; Pearson's Chi‐squared test is used for categorical variables; Fisher's exact test was used for categorical variables if any expected cell count < 5.

Abbreviation: NICU, neonatal intensive care unit.

^a^ Descriptive statistics reported; not adjusted for covariates or multiple testing. Instances where percentages of known outcomes do not total 100% are due to rounding.

^b^ Some hospital admissions and diagnoses cited multiple reasons.

In the screen‐guided care arm, there appeared to be a greater frequency of fetal growth restriction and preeclampsia at term. Participants in the routine care arm were more likely to have received antenatal corticosteroids (Table 5).

Excluding participants who were already receiving low‐dose aspirin at enrollment, 99 participants were initiated on low‐dose aspirin post‐enrollment (44 in the screen‐guided care arm and 55 in the routine care arm). Per provider decision as part of routine clinical management, progesterone was initiated after randomization in one lower‐risk participant in the screen‐guided care arm and in one participant in the routine care arm; both participants continued in the study.

### 3.3 Intervention bundle efficacy: Modified intent‐to‐treat analysis

The final mITT population, evaluated as specified in the protocol, included 4468 participants with complete outcome data (2143 screen‐guided care, 2325 routine care), and was balanced with regard to baseline maternal characteristics (Table S4). While inclusion in the mITT population did not require adherence to the treatment bundle after 24 weeks’ gestation for those at higher risk, 84% of higher‐risk participants accepting treatment completed at least 80% of nurse calls and medication doses after 24 weeks’ gestation.

Screen‐guided care significantly reduced composite neonatal morbidity (OR, 0.75; 95% CI, 0.62–0.91; *p* = 0.003) and shortened neonatal hospital length of stay by approximately 6% (IRR, 0.94; 95% CI, 0.90–0.97; *p* < 0.001) in this subpopulation. Notably, no patient identified by testing as higher risk in the mITT screen‐guided care arm delivered before 32 weeks of gestation. Tables S5 and S6 provide additional outcomes in the mITT population.

### 3.4 Safety

The medications within the bundled intervention were well tolerated. The most common adverse events were mild and related to vaginal progesterone (e.g., pruritus 0.40%, discharge 0.32%). One serious adverse event (0.04%) involved transient liver enzyme elevation with progesterone exposure, which resolved upon discontinuation of the medication.

Two neonatal deaths occurred in the routine care arm: one at 26 weeks’ gestation due to prematurity‐related complications, and another at 38 weeks due to subgaleal hemorrhage and hypoxic‐ischemic encephalopathy. No in‐hospital infant deaths occurred in the screen‐guided care arm.

## 4 DISCUSSION

In the PRIME trial, a targeted treatment bundle guided by mid‐second‐trimester maternal blood IGFBP4/SHBG screening was evaluated in women considered at low risk for singleton preterm birth. The findings demonstrate that biomarker‐informed, bundled care can deliver measurable improvements in important maternal and neonatal outcomes. Specifically, screen‐guided care was associated with reduced neonatal morbidity, shorter neonatal hospitalization, and a lower frequency of early preterm birth, underscoring the clinical potential of proactive risk stratification paired with targeted, individualized intervention.

Currently, preventive interventions implemented individually, such as progesterone [20, 43, 44], low‐dose aspirin [45, 46], or care management [21, 47] have positively impacted only a limited subset of pregnancies. This may stem from the multifaceted causes of preterm birth, suggesting that no single intervention is universally effective. This study employed a combination of preventive strategies to address the complexity of preterm birth, likely by addressing multiple pathways associated with an IGFBP4/SHBG‐defined higher‐risk state. Though the contribution of each intervention is challenging to pinpoint, in this study design, their collective application shows promise for targeting the diverse causes of preterm birth across larger populations through a biomarker‐based, screen‐guided care strategy.

Low‐dose aspirin and vaginal progesterone together exemplify how layered preventive strategies might benefit biomarker‐positive pregnancies. Each intervention appears to act on distinct yet interconnected pathways within the IGFBP4/SHBG‐defined higher risk state. Aspirin improves uteroplacental perfusion and limits platelet aggregation and cytokine‐mediated inflammation, thereby possibly supporting IGF‐driven nutrient transfer and counteracting inflammation‐induced suppression of SHBG [48]. Vaginal progesterone reinforces cervical integrity and modulates myometrial and immune activation, reducing premature cervical remodeling, lowering membrane fragility, and decreasing uterine contractility [49]. These multiple mechanisms of action provide a biologically coherent rationale for bundled intervention within a screen‐guided care framework and may underlie the population‐level improvements observed in this study. Knowledge of the IGFBP4/SHBG test result, when combined with structured care management and these interventions, represents a comprehensive strategy to address the multifactorial etiologies of preterm birth.

ITT analysis is commonly preferred in clinical trials because it maintains the benefits of randomization and may more closely reflect real‐world effectiveness. In this study, ITT analysis demonstrated significant improvements in additional maternal and neonatal outcomes across the entire randomized population.

The prespecified co‐primary outcomes were also analyzed in the mITT population to evaluate treatment efficacy, recognizing anticipated challenges with intervention uptake. Prior studies have shown that a substantial proportion of pregnant individuals may decline or discontinue interventions due to perceived risks and uncertain benefits [31, 50]. The mITT results showed further reductions in composite neonatal morbidity and mortality, and neonatal length of stay. Furthermore, in this population, not a single higher‐risk participant was delivered prior to 32 weeks’ gestation.

The study is strengthened by a multicenter design that incorporated a diverse population across community and university settings, enhancing generalizability, and reflecting real‐world applicability. Broad eligibility criteria included a large segment of the pregnant population, and the 7.8% preterm birth rate in the routine care arm, below the 2022 US singleton rate of 8.7% [51], confirmed that participants had reduced risk for preterm birth. Importantly, benefits were demonstrated even within this low‐risk cohort.

Outcome improvements were achieved using an IGFBP4/SHBG blood screening test combined with safe, low‐cost medications in an easily implemented intervention bundle. These findings suggest a strategy for reducing neonatal complications, particularly among those historically facing the highest risks and lowest benefits from current care models, including individuals from racial and ethnic minority populations and economically disadvantaged populations. Expanding access to this screening modality and care may also provide critical benefits to individuals in maternity care deserts, where the scarcity of pregnancy care and neonatal services poses substantial challenges to timely and effective intervention. By addressing these gaps with a mid‐second trimester screening test, earlier interventions could play a pivotal role in preventing harmful neonatal outcomes and improving health equity in underserved communities.

The benefits of IGFBP4/SHBG screen‐guided care observed in this study may extend beyond reductions in prematurity and immediate neonatal outcomes alone. These may include, but are not limited to, decreases in moderate neonatal complications, reductions in early‐life hospitalization and long‐term care costs, fewer parental work absences, lower rates of parental mental health disorders [52], and less strain on educational systems [53, 54]. Analysis of NICU days shows that screening and targeted treatment of just 4.2 low‐risk singleton pregnancies prevented one additional NICU day, illustrating a practical approach to risk stratification and targeted intervention. Additionally, screen‐guided care showed notable efficiency, with NICU admission data indicating a number needed to screen of 38.5 to prevent one admission. This number is substantially lower than the number needed to screen of 150 reported for the cervical length screening strategy endorsed by professional society guidelines, which recommend vaginal progesterone treatment in cases of cervical shortening, underscoring the superior efficiency of the proposed approach [55].

While the economic impact of these neonatal outcome improvements awaits a planned cost‐effectiveness analysis, the significant reductions in NICU admission, neonatal hospital length of stay, and neonatal morbidity—coupled with favorable screening and treatment metrics—highlight substantial clinical benefits and the potential for both immediate and long‐term cost savings.

An unanticipated finding in the screen‐guided care arm was the possible higher incidence of term fetal growth restriction and term preeclampsia. Rather than indicating an adverse shift, these results may instead reflect a redistribution of pregnancies with suboptimal placentation or inflammation toward later gestational ages at delivery. This interpretation is supported by the observed reduction in births before 32 and 35 weeks in the screen‐guided care arm. Conversely, the higher use of antenatal corticosteroids in the routine care arm is consistent with a greater proportion of deliveries before 34 weeks’ gestation, when corticosteroid administration is most commonly indicated.

Despite the trial's comprehensive scope, several limitations warrant consideration. In the open‐label screen‐guided care arm, where both patients and providers were aware of IGFBP4/SHBG test results, confounding factors may have influenced results. Providers may have altered management strategies—such as increasing surveillance or treatment—while patients could have adjusted behaviors to reduce perceived risks. However, modified management may be unlikely to achieve the study's observed outcomes due to the limits of current scalable treatments. In addition, an open‐label design was deemed necessary for several reasons: (1) Blinding the screen‐guided care arm was not readily feasible due to the implementation of the treatment bundle; (2) The inclusion of a placebo group would likely have limited enrollment due to the invasiveness of vaginally administered medications; and (3) A negative result in the screen‐guided care arm could alleviate stress and anxiety, which are associated with a higher risk of preterm birth. Blinding only the routine care arm preserved usual care practices, while the screen‐guided care arm was unblinded, thereby more closely reflecting real‐world clinical implementation.

As preterm birth complicates fewer than 10% of pregnancies, interventions designed to prevent preterm delivery are unlikely to produce measurable shifts in population‐level means or medians for gestational age at delivery. This effect was further attenuated in our study cohort, where the baseline preterm birth rate was lower than the US singleton average. Accordingly, the observed reduction in adverse neonatal outcomes occurred without a significant change in mean gestational age at delivery, reflecting the intervention bundle's targeted influence on early preterm birth rather than a generalized prolongation of all gestations. Most importantly, when gestational age was examined categorically, reductions were most evident at earlier gestational ages, when neonatal morbidity and mortality are greatest.

## 5 CONCLUSION

In summary, the PRIME trial demonstrates that mid‐second‐trimester blood IGFBP4/SHBG screening followed by a targeted intervention bundle significantly reduces neonatal morbidity and neonatal hospital length of stay compared with routine care and is associated with important reductions in early preterm birth. These findings, consistent across analytic populations, suggest that broader application of this screen‐guided care strategy could meaningfully improve maternal and neonatal outcomes, while possibly advancing a more equitable and effective model of obstetric care.

## AUTHOR CONTRIBUTIONS

Brian K. Iriye contributed to conceptualization. Michael Walker, Babak Shahbaba, and Jannik Godt contributed to data curation. Michael Walker, Babak Shahbaba, and Jannik Godt contributed to formal analysis. Brian K. Iriye, Michael Walker, Babak Shahbaba, and Jannik Godt contributed to methodology. Brian K. Iriye, John M. O'Brien, Christopher S. Ennen, Perry S. Barrilleaux, Jill A. Berkin, Anna Palatnik, Moeun Son, Cynthia Gyamfi‐Bannerman, Mollie McDonnold, Glenn R. Markenson, Anthony C. Sciscione, Joseph R. Biggio, Samuel Wolf, Scott A. Sullivan, and Stephen P. Pound contributed to resources. Brian K. Iriye contributed to supervision. Michael Walker, Babak Shahbaba, and Jannik Godt contributed to validation. Brian K. Iriye, Michael Walker, Babak Shahbaba, and Jannik Godt contributed to visualization. Brian K. Iriye, Michael Walker, Babak Shahbaba, and Jannik Godt contributed to writing—original draft. All authors contributed to writing—review & editing.

## CONFLICT OF INTEREST STATEMENT

B.K.I., J.M.O., C.S.E., P.J.B., J.A.B., A.P., M.S., C.G‐B., M.M., G.R.M., A.C.S., J.R.B., S.W., S.A.S., and S.P.P. report institutional funding from Sera Prognostics, Inc. (Sera) to support personnel costs related to conducting the study. J.M.O. was involved in studies of progesterone gel treatment for preterm birth prevention sponsored by a maker of progesterone gel. He was a principal investigator for studies published in 2011 and 2007. He has served on advisory boards and as a consultant for Watson, a company with a financial interest in marketing vaginal progesterone gel for preterm birth prevention. He and others are listed in a patent on the use of progesterone compounds to prevent preterm birth (USA Patent Number 7884093: Progesterone for the Treatment and Prevention of Spontaneous Preterm Birth). He has received other patents for devices to treat obstetric patients, including subpopulations at increased risk for preterm birth. He has not received any funds from a royalty agreement or licensing of any patent to date. C.G.‐B. reports grants from Mirvie outside the submitted work. J.R.B. reports grants from Mirvie outside of the submitted work and serves on a Regional Board of Directors for the March of Dimes. M.W., B.S., and J.G. report consulting fees paid from Sera during the conduct of the study. Sera provided medical writer support for manuscript preparation.

## ETHICS STATEMENT

The PRIME study was approved by a central institutional review board (Advarra, Columbia, MD, USA) and, where required, by local IRBs (details in Supporting Information). The trial was registered at ClinicalTrials.gov (NCT04301518) and conducted in accordance with CONSORT 2025 guidelines. Written informed consent was obtained in English or Spanish. A second consent was required for participants identified as higher‐risk in the screen‐guided care arm prior to receiving the intervention bundle.

## Supporting information



Supporting information

## Data Availability

Clinical protocols and the statistical analysis plan are available upon request to prime@sera.com; messages will be forwarded to the corresponding author and to Sera Prognostics, Inc. personnel. Data supporting the results described in this manuscript can be found in the article, Supporting Information, or obtained under controlled access by request to prime@sera.com. Given privacy restrictions on identifiable data, non‐commercial use will be evaluated upon request, subject to such restrictions and execution of a Data Transfer and Use Agreement with Sera Prognostics, Inc. Data will not be made available publicly or in any format that may violate a study participant's right to privacy. Requests for analysis code, data dictionaries, and study‐related forms can be sent to prime@sera.com.
