# Correction to “Neonatal impact of maternal biomarker screening for risk of preterm birth with targeted interventions (PRIME): A multicenter, randomized, controlled trial”

**DOI:** 10.1002/pmf2.70437

**Published:** 2026-09-06

Iriye, B. K., J. M. O’Brien, C. S. Ennen, Barrilleaux, P. S. Barrilleaux, J. A. Berkin, A. Palatnik, M. Son, et al. 2026. “Neonatal Impact of Maternal Biomarker Screening for Risk of Preterm Birth With Targeted Interventions (PRIME): A Multicenter, Randomized, Controlled Trial”. *Pregnancy* 2(1): e70202. https://doi.org/10.1002/pmf2.70202.

In Section 3.1, Participant Disposition, the intervention adoption rate by higher‐risk participants in the screen‐guided care arm was reported incorrectly. The article states: “Of these individuals, 57.7% (343/594) accepted and initiated the interventions before 24 weeks’ gestation.”

This reported rate is incorrect. Of the 594 participants identified at higher risk in this arm, 415 consented to receive interventions, 36 of whom did not initiate interventions prior to 24 weeks’ gestation as required. Upon recalculation, 63.8% (379/594) accepted and initiated the interventions before 24 weeks’ gestation. In the originally published version of this article, the Participant Disposition text should have read: “Of these individuals, 63.8% (379/594) accepted and initiated the interventions before 24 weeks’ gestation.”

In addition, the Supporting Information has been replaced to correct typographical/risk‐labeling errors in the Nurse Questionnaire in Supplementary Table 2: Weekly nurse call case report form, page 2. The corrected Nurse Questionnaire page supplied by the authors includes the following changes: item C2 response options specify “Yes (high risk)” and “No (low risk)”; item C3c includes the corrected mild contraction‐intensity responses, “Very mild: slight tightening (1–2/10) (low risk)”and “Mild: more intense tightening but not painful (3–4/10) (low risk)”; and item C4b response options specify “Yes (low risk)” and “No (moderate risk).”

These corrections do not alter the primary findings, interpretation, or conclusions of the article.

We apologize for these errors.

