# Supplementary material for: Neonatal impact of maternal biomarker screening for risk of preterm birth with targeted interventions (PRIME): A multicenter, randomized, controlled trial
Source: Pregnancy (Hoboken). 2026 Jan 6;2(1):e70202. doi: 10.1002/pmf2.70202 (PMC13344714; doi:10.1002/pmf2.70202)
Supplement: Supplementary file 1 [file PMF2-2-e70202-s001.pdf]

Supplementary Information

Supplement to: Iriye BK, O'Brien JM, Ennen, CS et al.,

Neonatal impact of maternal biomarker screening for risk of preterm birth with targeted interventions (PRIME): A multicenter, randomized, controlled trial

Supplementary Information

Neonatal impact of maternal biomarker screening for risk of preterm birth with targeted interventions (PRIME): A multicenter, randomized, controlled trial

Iriye BK, O'Brien JM, Ennen, CS et al.

Contents

1. Investigators 1
2. Statisticians 3
3. Study site research coordinator leads 4
4. PRIME Nurse Support Center (care management) 6
5. Data Coordinating Center 6
6. Data and safety monitoring board 7
7. Medical monitor 7
8. Institutional review board approvals 8
9. Care management protocol overview 11
10. Expanded methods 13
    - Trial oversight 13
    - Randomization and masking 13
    - Procedures 13
    - Statistical analysis 14
        - ITT and mITT estimands 14
        - Power and sample size analysis 14
        - Interim analysis of screen-guided care efficacy 15
        - Rationale for quantile-based efficacy analyses 15
        - Multiple imputation of missing data 16
        - Covariates and definition of reference arm 17
        - Adjustment for multiple hypothesis testing 17
        - Adjusting for neonatal death post-delivery 17
        - Tests of model assumptions 17
        - Number needed to screen 17
11. Table S1. Inclusion and exclusion criteria 18
12. Table S2. Weekly nurse call case report form 19
13. Table S3. Intent-to-treat analysis with multiple imputation for missing data. 22

14. Table S4. Baseline characteristics in the final modified intent-to-treat population. 23
15. Table S5. Additional interim and final outcomes in the mITT and ITT populations. 24
16. Table S6. Final primary and secondary outcomes, including adjustment for covariates, in the modified intent-to-treat population. 25
References 26

1. Investigators

Principal Investigator
Brian K. Iriye, MD
High Risk Pregnancy Center
Hera Women's Health
Las Vegas, NV

Investigators
John M. O'Brien, MD
Department of Obstetrics & Gynecology
University of Kentucky Health Care
Lexington, KY

Christopher S. Ennen, MD
Department of Obstetrics & Gynecology
University of Virginia School of Medicine
Charlottesville, VA

Stephen P. Pound, MD
Virginia Physicians for Women
North Chesterfield, VA

Perry S. Barrilleaux, MD
Department of Obstetrics and Gynecology
Louisiana State University Health Science Center
Shreveport, LA

Jill A. Berkin, MD
Department of Obstetrics, Gynecology, and Reproductive Science
Icahn School of Medicine at Mount Sinai
New York, NY

Anna Palatnik, MD
Department of Obstetrics and Gynecology
Medical College of Wisconsin
Milwaukee, WI

Moeun Son, MD, MSCI
Department of Obstetrics, Gynecology, and Reproductive Science
Yale University
New Haven, CT
Department of Obstetrics and Gynecology
Weill Cornell Medical College
New York, NY

(continued on next page)

1. Investigators (continued)

Cynthia Gyamfi-Bannerman, MD, MS
Department of Obstetrics, Gynecology, and Reproductive Sciences
University of California San Diego
La Jolla, CA USA

Mollie McDonnold, MD
Austin Maternal-Fetal Medicine
St. David's Women's Center of Texas
Austin, TX

Glenn R. Markenson, MD
Department of Obstetrics & Gynecology
Boston University Chobanian and Avedisian School of Medicine
Boston, MA

Anthony C. Sciscione, DO
Department of Obstetrics and Gynecology
ChristianaCare Health System
Department of Maternal-Fetal Medicine
Delaware Center for Maternal Fetal Medicine
Newark, DE

Joseph R. Biggio, Jr., MD, MS
Department of Obstetrics and Gynecology
Ochsner Health
New Orleans, LA

Samuel Wolf, DO
Emerald Coast OBGYN Clinical Research
Panama City, FL, USA

Scott A. Sullivan, MD, MSCR
Department of Obstetrics and Gynecology
Inova Health
Fairfax, VA, USA

2. Statisticians

Leah Bednarek, MS
Foster Consulting Group
Sellersville, PA

Jason Connor, PhD
Confluence Stat, LLC
Cooper City, FL

Jannik Godt
JGConsult
Nærum, Denmark

Bernet Kato, PhD
Carelon Research
Wilmington, DE

Gayatri Ranganathan, MS
Carelon Research
Wilmington, DE

Babak Shahbaba, PhD
Department of Statistics
UCI Data Science Initiative
University of California, Irvine
Irvine, CA

Maxwell Van Over, MPH
Carelon Research
Wilmington, DE

Michael Walker, PhD
Walker Bioscience
Carlsbad, CA

3. Study site research coordinator leads

Jennifer Somdahl, BSN, RN
High Risk Pregnancy Center
Las Vegas, NV

Wendy Whitley, BSN, RN
University of Kentucky Health Care
Lexington, KY

Amanda Urban, CRC
University of Virginia School of Medicine
Charlottesville, VA

Madison Harvell, CRC
Virginia Physicians for Women
North Chesterfield, VA

Shannon Phillips, BSN, RN
Louisiana State University Health Science Center
Shreveport, LA

Monica Patel, CRC
Icahn School of Medicine at Mount Sinai
New York, NY

Eleanor Saffian, RNC-OB
Medical College of Wisconsin
Milwaukee, WI

Jenna Gartner, BSN, RN
Yale University
New Haven, CT

Beatriz Valeiro Gonzalez, CRC
University of California San Diego
La Jolla, CA

Heather Azarmehr, MSN, RN
Austin Maternal-Fetal Medicine
Austin, TX

Gabriela Mateo, CRC
Boston University Chobanian and Avedisian School of Medicine
Boston, MA

(continued on next page)

3. Study Site Research Coordinator Leads (continued)

Natalie Onesi, CRC
ChristianaCare Health System
Delaware Center for Maternal Fetal Medicine
Newark, DE

Paige Delaune, CRC
Ochsner Health
New Orleans, LA, USA

Lauren Green, CRC
Emerald Coast OBGYN Clinical Research
Panama City, FL

Chinacherem Chinaka, MPH, MSN, RN
Inova Health
Fairfax, VA

Michelle Crawford, MSN, FNP
High Risk Pregnancy Center
Las Vegas, NV

4. PRIME Nurse Support Center (care management)

Weekly telephonic outreach for consenting study participants with biomarker-ascertained higher preterm birth risk was provided by:

- Maureen Brennan, RNC, BSN
- Jennifer Baker, RN, BSN
- Caroline Granfield, RN
- Jenny Stimac, CNM, MS
- Pamela West, RN, BSN

5. Data Coordinating Center

Carelon Research
Wilmington, DE

Principal Scientist
Eric Stanek, PharmD

Project Management

- Ashley Magnavita, MPH
- Rachna Kumar, MD
- Ashley Gursoy

Biostatistics

- Bernet Kato, PhD
- Gayatri Ranganathan, MS
- Maxwell Van Over, MPH

Data Management/Collection

- Kinjal Patel, MS
- Mark Paullin, MS
- Rebecca Conto, MA
- Joseph Singer, MD
- Mahitha Kothapalli, MPH, MSPM

Clinical Research Associate

- Adrienne Bodner, BA

6. Data and safety monitoring board

Chair
Christopher Robinson, MD, MSCR, FACOG
Charleston Maternal Fetal Medicine
Charleston, SC

Members
Jacquelyn Knupp Patterson, MD, MPH
UNC Health
Chapel Hill, NC

Michelle Secic, MS
Secic Statistical Consulting, Inc.
Cleveland, OH

7. Medical monitor

Richard Porreco, MD
Obstetrix Medical Group of Colorado
Retired
Denver, CO

8. Institutional review board approvals

The institutional review boards (IRBs) of all institutions participating in the study gave ethical approval for this work. The following is a listing of PRIME (NCT04301518) study approvals.

Advarra, Inc. (Columbia, MD) served as a central IRB for the following sites:

Austin Maternal-Fetal Medicine
Austin, TX
PI: Mollie McDonnold, MD
IRB/Approval #: Pro00045517
FWA: 00023875

Corewell Health William Beaumont University Hospital
Royal Oak, MI
PI: Zeynep Alpay-Savasan, MD
IRB/Approval #: Pro00045517
FWA: 00023875

Emerald Coast Obstetrics and Gynecology
Panama City, FL, USA
PI: Samuel Wolf, DO
IRB/Approval #: Pro00045517
FWA: 00023875

High Risk Pregnancy Center
Las Vegas, NV
PI: Brian K. Iriye, MD
IRB/Approval #: Pro00045517
FWA: 00023875

Icahn School of Medicine at Mount Sinai
New York, NY
PI: Jill A. Berkin, MD
IRB/Approval #: Pro00045517
FWA: 00023875

Inova Health
Fairfax, VA
PI: Scott A. Sullivan, MD, MSCR
IRB/Approval #: Pro00045517
FWA: 00023875

(continued on next page)

8. Institutional Review Board Approvals (continued)

Ochsner Health
New Orleans, LA
PI: Joseph R. Biggio, Jr. MD, MS
IRB/Approval #: Pro00045517
FWA: 00023875

Ochsner Louisiana State University Health Shreveport
Shreveport, LA
PI: Perry S. Barrilleaux, MD
IRB/Approval #: Pro00045517
FWA: 00023875

OhioHealth
Columbus, OH
PI: Mona R. Prasad, DO, MPH
IRB/Approval #: Pro00045517
FWA: 00023875

University of California San Diego
La Jolla, CA
PI: Cynthia Gyamfi-Bannerman, MD
IRB/Approval #: Pro00045517
FWA: 00023875

University of Texas Medical Branch
Galveston, TX
PI: George R. Saade, MD
IRB/Approval #: Pro00045517
FWA: 00023875

University of Virginia School of Medicine
Charlottesville, VA
PI: Christopher S. Ennen, MD
IRB/Approval #: Pro00045517
FWA: 00023875

Virginia Physicians for Women
North Chesterfield, VA USA
PI: Stephen P. Pound, MD
IRB/Approval #: Pro00045517
FWA: 00023875

(continued on next page)

8. Institutional Review Board Approvals (continued)

Yale University
New Haven, CT
PI: Moeun Son, MD
IRB/Approval #: Pro00045517
FWA: 00023875

Study site-specific IRBs:

Boston University Institutional Review Board
Boston, MA
PI: Glenn R. Markenson, MD
IRB/Approval #: H-42137
FWA: 00000301

ChristianaCare Institutional Review Board
Newark, DE
PI: Marwan Ma'Ayeh, MD
IRB/Approval #: CCC#43023
FWA: 0006557

Cleveland Clinic Institutional Review Board
Cleveland, OH
PI: Edward K. Chien, MD
IRB/Approval #: 21-828
FWA: 00005367

Medical College of Wisconsin Institutional Review Board
Milwaukee, WI
PI: Anna Palatnik, MD
IRB/Approval #: PRO41709
FWA: 00000820

University of Kentucky Office of Research Integrity IRB, RDRC
Lexington, KY
PI: John M. O'Brien, MD
IRB/Approval #:63127
FWA: 00005295

9. Care management protocol overview

Treatment of Women at Higher Risk for Preterm Birth (biomarker test score >15%)
Consenting participants in the biomarker-ascertained "higher-risk" group will be provided the protocol-defined interventions after completing the intervention protocol informed consent process and providing written informed consent. Those who provide consent must begin interventions prior to $24^{0/7}$ weeks' gestation:

1. Care Management: Participants will receive weekly nurse support once-weekly (Table S2). Contact will consist of a weekly telephone call beginning the week after the participant provides consent for interventions. Weekly calls will continue until delivery or through the 37th week of gestation to capture intervention compliance through $36^{6/7}$ weeks gestation, whichever occurs first. Weekly contact will accomplish the following goals:
    - Review symptoms of preterm labor and premature rupture of membranes using a standardized survey of symptoms with pre-specified indicators to trigger a clinical evaluation, such as decreased fetal movement, fever, contractions, signs or symptoms of preeclampsia and/or vaginal bleeding and discharge.
    - Confirm and document compliance with study medication use.
    - Provide educational content regarding PTB and when to seek immediate care (i.e. go to the hospital).
    - Provide appointment monitoring (address transportation issues, missed appointments, etc.).
    - Allow for communication from participant to intervention team to address any questions or concerns on the part of the participant. Any health concerns not addressable by the support nurse, e.g. health conditions affecting pregnancy such as diabetes, hypertension, smoking, etc., will be addressed by the intervention team upon notification by the research nurse.

2. Progesterone: Participants will be prescribed 200 mg of micronized progesterone to be self-administered intravaginally every night from start of intervention (<$24^{0/7}$ weeks' gestation) through $36^{6/7}$ weeks' gestation or delivery, whichever occurs first. Progesterone will be obtained by the participant via a study-specific debit card provided to the participant.

3. Low-dose aspirin: Participants will be provided low-dose aspirin therapy (81 mg orally daily) from start of intervention (<$24^{0/7}$ weeks' gestation) through $36^{6/7}$ weeks' gestation or delivery, whichever occurs first. Aspirin will be provided by the research site to the participant.

4. Cervical length monitoring: Participants will receive additional monitoring via transvaginal ultrasound for cervical length measurement at two subsequent study visits:
    - First follow-up ultrasound: between $21^{0/7}$ and $23^{6/7}$ weeks' gestation.
    - Second follow-up ultrasound: between $26^{0/7}$ and $28^{6/7}$ weeks' gestation.
    - Participants will already be offered the standard of care treatment for shortened cervix in the form of daily vaginal progesterone.

5. Cerclage: If cervical length is less than or equal to 10 mm prior to $24^{0/7}$ weeks, placement of a cerclage will be recommended for the participant. Final determination of cerclage placement will be made by physician and participant on a per-participant basis. Placement of cerclage is not a requirement for the study.

9. Care management protocol overview (continued)

All participants with higher-risk scores who consent to the interventional component of the protocol will be given a study summary sheet to keep on their person that explains their participation in the study, the tenets of their intervention plan, and instructions to notify the study investigator on-call for questions of suspected preterm labor. The study investigator will ensure all tenets of the treatment protocol are consistently adhered to in the management of the participant throughout the pregnancy.

Antenatal corticosteroids should be administered as per standard of care guidelines for any woman who develops symptoms indicating that she is at higher risk of preterm delivery and in whom the clinician believes there is a high likelihood of delivering in the ensuing 7 days.[1]

For individuals participating in study interventions, if their health insurance coverage is discontinued during the duration of study related activities, the participant's active follow-up will not be altered.

10. Expanded methods

Trial oversight

An independent data and safety monitoring board. provided analysis supervision. Carelon Research (Wilmington, DE) handled data collection and primary and secondary outcome analysis in the modified intent-to-treat (mITT) population. Independent statisticians confirmed all statistical results. Two additional statisticians independently verified the statistical code and confirmed primary, secondary, and NICU admission analyses. The final manuscript content was controlled by the principal investigator with editorial input by coauthors.

Randomization and masking

The independent data coordinating center (Carelon Research, Wilmington, DE, USA) provided computer-generated simple randomization of participants in a 1:1 ratio to one of two parallel arms, the routine care or screen-guided care arm, which was executed following receipt of each analyzable sample but prior to testing. The random sequence was not available before allocation. This open-label study allowed participants and investigators to be aware of arm allocation after assignment and throughout the trial. To ensure integrity, all participants, investigators, and personnel involved in neonatal care, deliveries, and data collection remained blinded to test results from the routine care arm throughout the study. Investigative teams were often not the caregiving providers beyond implementation of the treatment bundle or notification of results in the screen-guided care arm.

Interim analyses were also blinded except for the statisticians at the data coordinating center, who conducted the analyses, and a separate blinded statistician unaffiliated with the study, who presented results to the DSMB during closed sessions.

Final analyses of the primary and secondary endpoints were conducted by the data coordinating center under blinded conditions. Results were first presented to the DSMB in a closed session for review before unblinding outcome results to investigators, participants, and the study sponsor.

Procedures

Blood samples were collected from all participants, and the IGFBP4/SHBG ratio assessed via liquid chromatography-mass spectrometry in an accredited clinical laboratory (Sera Prognostics, Inc., Salt Lake City, UT, USA), as described previously.[2] Test results in the routine care arm were blinded. Participants randomized to the screen-guided care arm were classified as "higher-risk" (Figure 1) if their algorithm-based IGFBP4/SHBG-scored preterm birth risk was ≥15%.[3] The remaining screen-guided care arm participants were referred to as "not higher risk."

Participants in the routine care arm and the not-higher-risk group of the screen-guided care arm received routine obstetric care. Screen-guided care arm participants at higher risk underwent a second consent before receiving a preterm birth intervention bundle, beginning before $24^{0/7}$ weeks' gestation. As part of the intervention, participants agreed to self-administer intravaginal progesterone (200 mg) nightly until $36^{6/7}$ weeks' gestation or delivery, whichever came first. Low-dose aspirin (81 mg orally daily) was prescribed to the higher-risk screen-guided care arm participants for the same duration. Weekly nurse support was provided to these participants as a structured telephone call through the 37th week of gestation or delivery, whichever occurred first, to capture intervention compliance and complications of pregnancy through $36^{6/7}$ weeks' gestation. Patient-reported information provided during the telephone call was used to complete a weekly nurse call case report form (Supplementary Table 2). This weekly contact achieved five goals: (1) It provided a review of symptoms of preterm labor and

premature rupture of membranes using a standardized survey of symptoms with pre-specified indicators to trigger a clinical evaluation, such as decreased fetal movement, fever, contractions, signs or symptoms of preeclampsia and/or vaginal bleeding and discharge. (2) The nurse contact confirmed and documented adherence to study medication use. (3) Nurse contact provided educational content regarding preterm birth symptoms and when to seek immediate care. (4) Nurses provided appointment monitoring (addressing transportation issues, missed appointments, etc.). (5) Nursing contact facilitated communication between participant and their obstetric care team to address any questions or concerns on behalf of the participant. Any health concerns not addressable by the support nurse, e.g., health conditions affecting pregnancy such as diabetes, hypertension, smoking, etc., were to be addressed by the participant's obstetric care team upon notification by the research nurse.

Statistical analysis

ITT and mITT estimands

Two estimands were used to evaluate treatment efficacy: the treatment policy estimand, defined in the FDA Guidance for Industry E9(R1) Statistical Principles for Clinical Trials: Addendum on Estimands and Sensitivity Analysis,[4] which assessed effects in the intent-to-treat (ITT) population regardless of intervention acceptance; and the trial product estimand, which assessed effects in the mITT population excluding higher-risk participants who declined intervention.

The treatment policy estimand measures average population effects of primary interest to regulatory authorities. The trial product estimand estimates expected outcomes for participants who undergo testing and accept interventions as intended.

Power and sample size analysis

Sample size was based on simulations and analyses using NCSS PASS 2020[5] to ensure adequate power for both co-primary outcomes.

Power and sample size for this study were affected by nuisance factors including the proportion of participants receiving IGFBP4/SHBG risk scores at or above the threshold of 15% risk (the study screen positive rate [SPR]), the proportion of participants in the Higher Risk group accepting and adhering to interventions (compliance), and the rate at which participants and their neonates remain in the study (retention). For the purpose of power and sample size estimation, and based on prior intervention study test data,[6,7] we assumed that 29% of participants in the screen-guided care arm will have IGFBP4/SHBG risk scores at or above the threshold of 15% risk (the SPR).

Power and sample size are also affected by the study population's baseline level of risk of adverse neonatal outcomes related to prematurity, the test's sensitivity in identifying at-risk pregnancies, and the efficacy of the interventions as implemented in the study. Literature indicates that the effect of treatment is not constant across gestational age at birth.[8-13] Thus, there is no single value of the effect size in terms of proportion of participants with NMI ≥3 or neonatal length of hospital stay. Rather, the effect size is larger for participants with pre-treatment potential for earlier gestational age at birth, while the effect size is reduced for participants with later pre-treatment potential gestational age at birth.

For the first co-primary endpoint, reduction of severe composite morbidity/mortality, a range of possible expected effect sizes was inferred from the simulations. Based on the simulations, the proportion of participants with NMI ≥3 was expected to be between 0.018 and 0.013 in the screen-guided care arm and near 0.031 in the routine care arm. Assuming these proportions, a two-sided Fisher Exact test, and

the interim analysis schedule outlined below, the required sample size is 2800 participants with outcomes in the screen-guided care arm and 2800 participants with outcomes in the routine care arm (5600 total) to provide power of 0.65-1.0 (NCSS PASS 2020, module "Group-Sequential Tests for Two Proportions").

For the second co-primary endpoint, length of neonatal hospital length of stay from birth up to neonatal discharge, assessed by Cox proportional hazards regression with stratification, the hazard ratio based on simulations was expected to be between 1.44 and 1.58. Assuming these hazard ratios and the interim analysis schedule outlined below, the required sample size is 2800 participants with outcomes in the screen-guided care arm and 2800 participants (5600 total) with outcomes in the routine care arm to provide a power of 0.9-1.0 (NCSS PASS 2020, modules "Group-Sequential Tests for Two Hazard Rates" and "Tests for Two Survival Curves Using Cox's Proportional Hazards Model").

Based on these simulations and projected loss to follow-up of 16%, the target sample size was 6500 participants.

<u>Interim analysis of screen-guided care efficacy</u>
The PRIME trial statistical analysis plan (SAP) prespecified an interim analysis of screen-guided care efficacy to be conducted in the mITT population when approximately 1400 eligible participants per arm had delivered with outcome data. The number of 1400 per arm (2800 combined) was 50% of the 5600 total for the final primary analysis. Interim analysis was assessed by the independent DSMB without unblinding the study sponsor or investigators, and later confirmed by a second independent statistician.

Boundary conditions were calculated using NCSS PASS 2020.[14] The O'Brien-Fleming[15] nominal *p* values for stopping trial enrollment were 0.0056 for the interim efficacy analyses and 0.0482 for the final analyses. Type I error in testing the two co-primary endpoints was controlled using Holm's method,[16] requiring that the co-primary outcome having the lowest *p* value reach <0.0028 to be significant at the interim and <0.0241 at the final analysis. The second co-primary outcome was then tested at the full alpha of 0.0056 at the interim and 0.0482 at the final analysis. Interim analyses were adjusted for pre-enrollment aspirin use and parity. At the interim, COVID-19 status did not differ by arm, and was not used as a covariate in analyses.

The primary analyses used ordinal logistic regression for neonatal morbidity and mortality and Cox regression in the quantile of longest stays for neonatal length of hospital stay. The secondary analyses used Cox regression in the quantile of earliest births for gestational age at birth and Cox regression in the quantile of longest stays for NICU length of stay. Per the SAP, the quantile for time-to-event analyses was set at 1.2 times the preterm birth rate in the routine care arm. With a preterm birth rate of 7.8%, the quantile was calculated to be 9.4% and applied equally to both arms. The extreme 9.4% of an outcome was calculated individually for neonatal length of hospital stay, NICU length of stay and gestational age at birth, as opposed to selecting a cohort of participants according to the extreme 9.4 percentile of one outcome and analyzing that same cohort for the other two outcomes.

<u>Rationale for quantile-based efficacy analyses</u>
Detecting changes in neonatal outcomes across a broad population with low overall risk for preterm birth presents analytic challenges. The bundled interventions were specifically designed to improve outcomes among the approximate 10% of neonates typically born preterm and were not expected to influence outcomes for the remaining 90% of term births. Accordingly, analyses of neonatal hospital and

NICU length of stay focused on the longest-stay quantiles, while gestational age at birth was evaluated using the earliest-birth quantile.

In clinical trials, statistical methods must distinguish true intervention effects from background variation while detecting clinically meaningful outcome shifts. In studies of preterm birth, quantile analysis offers distinct advantages over analyses restricted to either preterm deliveries or the entire population. Limiting analyses to preterm births can reduce statistical power when one arm has fewer preterm deliveries, introduce imbalance in group sizes, and overlook benefits from shifting births from preterm to early term. Conversely, whole-population analyses may dilute effects, as interventions typically impact only a small subset of patients who deliver preterm, making changes in overall means or medians difficult to detect.

Quantile-based analyses address these limitations by examining a consistent proportion of participants in each arm—specifically those with the earliest births and longest hospital stays—who are most likely to benefit from intervention. Selecting a quantile just above the control arm's preterm birth rate captures shifts in gestational age across both preterm and early term births, while excluding most term births unlikely to be affected. This approach also isolates changes in hospital stay duration among the most impacted neonates, minimizing confounding from brief, uncomplicated term stays.

By focusing on the population most likely to benefit and maintaining balanced group comparisons, quantile analysis enhances statistical efficiency and improves the detection of clinically meaningful effects. It can reveal outcome differences that are obscured when only measures of central tendency are used.[17,18] For example, in an obstetrical study of periodontal disease treatment, standard analyses showed no change in mean birthweight due to offsetting effects: an increase in higher birth weights linked to gestational diabetes and a reduction in lower birthweights from decreased preterm birth and growth restriction.[19] Quantile analysis reveals these divergent shifts, highlighting improvements missed by mean-based methods.

<u>Multiple imputation of missing data</u>
We performed a series of sensitivity analyses of the primary and secondary outcomes. These sensitivity analyses evaluated: (1) the robustness of the results when examined using alternative statistical models; and (2) the robustness of the results when analyzed using multiple imputation methods assuming missing at random (MAR) and then missing not at random (MNAR).

Multiple imputation was performed to account for missing outcomes among participants and included all randomized and enrolled participants. The models included the imputed outcome variable, aspirin use before pregnancy, parity, hypertension, diabetes, and confirmed COVID. The imputation step used Fully Conditional Specification (FCS),[20] with FCS logistic for binary variables and FCS regression predictive mean matching for quantitative and ordered categorical variables. For the missing-at-random analyses, missing values were imputed using predictions based on the observed outcome data from the same treatment group. For the missing-not-at-random analyses, missing values were imputed using predictions based on the observed outcome data from the routine care group ("jump to reference"). The analysis step used the same method for each outcome (ordinal logistic regression, Poisson regression, zero-inflated Poisson regression) as used for the analyses not using multiple imputation. The multiple imputation analyses were performed using SAS PROC MI and SAS PROC MIANALYZE.[20]

Covariates and definition of reference arm

Results for the final analyses were adjusted for the prespecified covariates pre-enrollment aspirin use, parity, and confirmed COVID-19 status (determined via FDA-authorized SARS-CoV-2 diagnostic testing post-enrollment), unless otherwise indicated. For all regression models, other than for gestational age at birth, reference arms were chosen such that ratios below one indicates improved outcomes in the screen-guided care arm compared to the routine care arm. For regression of gestational age at birth, the reference arm was selected such that a hazard ratio above one indicates prolongation of gestation.

Adjustment for multiple hypothesis testing

Adjustment for multiple hypothesis testing used Holm's method[16] for the primary analyses. *p* values were not adjusted for secondary, exploratory, or sensitivity analyses. The 95% confidence intervals for individual endpoints do not account for the family-wise error rate.

Adjusting for neonatal death post-delivery

Neonatal death from extreme prematurity, while classified as mortality, artificially shortens hospital stay and may bias analyses by underestimating neonatal and NICU length of stay relative to survival outcomes. To account for death when assessing time-to-event outcomes, such as hospital or NICU length of stay, a variety of statistical methods have been reported in the literature. These include excluding patients who die from the analyses, using the time to death as the length of stay, using competing risk survival models (when deaths constitute a sufficiently large proportion of outcomes), imputing a value such as the mean or median length of stay, or a worst case imputation, in which the maximum observed length of stay is imputed in the case of death.[21-23] Because only two deaths occurred in this study, the number was insufficient for a meaningful competing-risks survival analysis. We present two alternative analyses: (i) use the time to death as the length of stay, and (ii) use a worst-case imputation, in which the maximum length of NICU stay plus 1 day is imputed in the case of death.

Tests of model assumptions

Potential violations of the proportional hazards assumptions in the interim and final analyses were evaluated using Schoenfeld residuals (for Cox models).[24] If assumptions were violated, the analysis was repeated using the Brown-Mood median test[25] and restricted mean survival time for neonatal length of hospital stay, NICU length of stay, and gestational age at birth. The proportional odds assumption for ordinal logistic regression was evaluated with the Brant test[26] for neonatal morbidity and mortality.

Number needed to screen

The number needed to screen (NNS) to prevent one NICU day was calculated as the reciprocal of the mean difference in NICU days, which included zero-day stays for any neonate not admitted to the NICU. The NNS to prevent one NICU admission was calculated as the reciprocal of the absolute risk reduction of NICU admissions. Calculations were based on the ITT population. Confidence intervals for NNS values were computed by Poisson regression. *p*-values for the difference in NICU days or NICU admissions were computed by Wilcoxon rank-sum test or Pearson's chi-squared test, respectively and were not adjusted for multiple comparisons.

11. Table S1. Inclusion and exclusion criteria

**Inclusion criteria**	
1.	Participant is 18 years of age or older.
2.	Participant is willing and able to provide informed consent and comply with intervention if applicable.
3.	Participant gestational age is currently within 18 weeks 0 days and 20 weeks 6 days' using best estimated due date.
4.	This is a singleton intrauterine pregnancy.
5.	Participant has no signs and/or symptoms of preterm labor and has intact membranes.
6.	Participant has had a second-trimester anatomic ultrasound, including evaluation of cervical length, completed by the date of enrollment, but no earlier than 18 weeks 0 days' gestation.
7.	In the opinion of the Investigator, the participant's delivery and neonatal data will be accessible for data collection for the site.
**Exclusion criteria**	
1.	Previous history of spontaneous delivery before 37 weeks of gestation.
2.	Cervical length <25 mm in 2nd trimester at time of enrollment.
3.	Taking progesterone or a progesterone derivative after 13 weeks 6 days' gestation.
4.	History of selective reduction in current gestation / known vanishing twin in current gestation.
5.	Major fetal anomaly / chromosomal or genetic abnormality.
6.	Placenta accreta spectrum disorder.
7.	Placenta covers internal os by more than 2.5 cm at time of second trimester ultrasound (18 weeks 0 days – 20 weeks 6 days' gestation; confers high risk for term placenta previa).
8.	Vaginal bleeding >13 weeks 6 days' gestation.
9.	One or more of the following uterine risk factors: fibroids >5.0 cm, uterine malformation, history of classical cesarean, history of uterine surgery with trans-myometrial penetration (excluding previous cesarean).
10.	Planned cesarean or induction <37 weeks of gestation.
11.	Cerclage or pessary placement prior to enrollment.
12.	Participant on heparin or low molecular weight heparin since last menstrual period.
13.	Current polyhydramnios.
14.	Use of illicit drugs in the current pregnancy that increase preterm birth: cocaine, methamphetamine, opioid use disorder.
15.	Allergic to aspirin or history of gastrointestinal bleeding with aspirin use.
16.	Allergic to peanuts and/or peanut oil used in exogenous progesterone formulation.
17.	Participation in any other interventional research studies during the current pregnancy.
18.	Participant has tested positive for COVID-19 via a U.S. Food and Drug Administration-authorized diagnostic test for SARS-CoV-2 within the ten days prior to sample collection for IGFBP4/SHBG preterm birth risk stratification.
19.	Participant has or has had moderate to severe COVID-19 symptoms per American College of Obstetricians and Gynecologists/Society for Maternal-Fetal Medicine (ACOG/SMFM) Outpatient Assessment and Management for Pregnant Women with Suspected or Confirmed Novel Coronavirus (COVID-19)23 in an emergency room or hospital setting since the last menstrual period reference.
20.	Participant has a chronic medical disease requiring intensive medical surveillance and may require preterm delivery to include lupus, chronic lung disease on oxygen replacement, cardiac disease with high risk of maternal mortality including Marfan syndrome with dilated aortic root, significant pulmonary hypertension, neuromuscular disease at risk for pulmonary insufficiency such as myotonic dystrophy, renal failure on dialysis, and uncontrolled or poorly controlled hyperthyroidism.

12. Table S2. Weekly nurse call case report form

**Nurse Support Calls and Compliance Visit (page 1)**<br>**Weekly Nurse Support**			
**Section A: Key Identifying Information**			
**A1. Participant ID**	(remote value)		
A2. Weekly Nurse Call Number	☐		(format xx)
Check if call not performed; unable to contact participant	☐		
A3. Date last phone call attempt was made for this week's call:	☐		(DD-MM-YYYY)
Ref: Gestational age at the time last call was made	(auto-calculated)		(format xx.xx)
A4. Date of call:	☐		(DD-MM-YYYY)
Ref: Gestational age at the time of call	(auto-calculated)		(format xx.xx)
A5. Time of call:	☐		(HH24:MI)
**Section B: Weekly Update**			
B1. Did the participant have a hospital visit since last contact due to symptoms of preterm labor/birth?	☐<br>☐	Yes<br>No	
B2. Did the participant have any moderate to severe COVID-19 symptoms per the ACOG/SMFM[a] *Outpatient Assessment and Management for Pregnant Women with Suspected or Confirmed Novel Coronavirus (COVID-19)*[b] since the last contact?	☐<br>☐	Yes<br>No	
B3. Did the participant have a confirmed diagnosis of COVID-19 via an FDA-authorized diagnostic test since the last contact?	☐<br>☐	Yes<br>No	
B4. Did the participant have any other illnesses since the last contact?	☐<br>☐	Yes<br>No	
B5. Did the participant have any signs and symptoms of preeclampsia since last contact?	☐<br>☐	Yes<br>No	
B5a. Which of these symptoms did the participant have?	☐<br>☐<br>☐<br>☐<br>☐	Headache<br>Swelling<br>Blurred vision or spots in front of your eyes<br>Upper right quadrant pain/epigastric pain<br>None of the above	
B6. Did the participant have any accidents, falls, or traumas since the last contact?	☐<br>☐	Yes<br>No	
B6a. If yes, explain:	☐		
B7. Did the participant take to smoking/vaping/nicotine use since the last contact?	☐<br>☐	Yes<br>No	Whether new or continued use, please indicate "yes" if the participant has smoked, vaped, or used nicotine since the last contact.
B8. Has the participant complied with daily dosing of progesterone since last contact?	☐<br>☐<br>☐	Yes<br>No<br>Not Applicable	
B8a. How many doses did the participant miss since the last nursing contact?	☐		
B9. Has the participant complied with daily dosing of aspirin since last contact?	☐<br>☐<br>☐	Yes<br>No<br>Not Applicable	
B9a. How many doses did the participant miss since the last nursing contact?	☐		(format xx)

**Nurse Support Calls and Compliance Visit (page 2)**<br>**Weekly Nurse Support**		
**Section C: Questionnaire**		
Instructions: The highest level of risk assessed for any question will also be the overall risk for the participant. Bracketed text are the assigned risk levels for assessment.		
C1. How would you describe the movement of your baby?	☐<br>☐<br>☐<br>☐	Normal (does not alter risk)<br>Increased (does not alter risk)<br>Decreased (recommend kick counts, high risk)<br>Absent (recommend kick counts, high risk)
C2. Have you had a fever (>100 degrees F) since our last contact?	☐<br>☐	Yes<br>No
C3. Have you had any episodes of contractions since our last contact?	☐<br>☐	Yes<br>No
C3a. How often are you having an episode of contractions?	☐<br>☐<br>☐	One per day (low risk)<br>2-3 times per day (moderate risk)<br>>3 times per day (high risk)
C3b. During the episode, how many contractions do you have per hour?	☐<br>☐<br>☐<br>☐	<1 per hour (low risk)<br>1-2 per hour (low risk)<br>3-5 per hour (moderate risk)<br>6 or more per hour (high risk)
C3c. During the episode, how intense are the contractions?	☐<br>☐<br>☐<br>☐<br>☐	Very mild: slight tightening (1/2-10) (low risk)<br>Mild: more intense tightening but not painful (3/4-10) (low risk)<br>Moderate: light cramping like a period (5-6/10) (moderate risk)<br>Strong cramping and unable to talk during contractions due to discomfort (7-8/10) (high risk)<br>Severe pain (9-10/10) (high risk)
C3d. Do the contractions stop when you rest or drink fluids?	☐<br>☐	Yes (low risk)<br>No (moderate risk)
C4. Have you experienced any vaginal bleeding since our last contact?	☐<br>☐	Yes<br>No
C4a. How long have you had vaginal bleeding?	☐<br>☐<br>☐<br>☐	<24 hours (does not alter risk)<br>1-2 days (does not alter risk)<br>3-4 days (does not alter risk)<br>>4 days (does not alter risk)
C4b. Did bleeding begin within 24 hours of intercourse?	☐<br>☐	Yes<br>No
C4c. How would you describe the color of the bleeding?	☐<br>☐<br>☐	Pink-tinged (low risk)<br>Dark brown (moderate risk)<br>Bright red (high risk)
C4d. How would you describe the amount of bleeding?	☐<br>☐<br>☐	Spotting: 1 to 2 drops on underwear or streaks in mucous (does not require a pad (low risk)<br>Moderate: like a light period (requires 1-2 pads per day) (moderate risk)<br>Heavy: greater than or equal to a heavy period (requires >2 pads per day) (high risk)
C5. Have you experienced a change in your vaginal discharge since your last contact?	☐<br>☐	Yes<br>No
C5a. How long have you had vaginal discharge?	☐<br>☐<br>☐<br>☐	<24 hours (does not alter risk)<br>1-2 days (does not alter risk)<br>3-4 days (does not alter risk)<br>>4 days (does not alter risk)
C5b. How would you describe your discharge?	☐<br>☐<br>☐	Mucous (moderate risk)<br>Bloody (high risk)<br>Other (moderate risk)

Nurse Support Calls and Compliance Visit (page 3)
Weekly Nurse Support

**Section C: Questionnaire (continued)**	
C6. Have you experienced any leaking of watery, clear fluid since our last contact?	☐ Yes<br>☐ No
C6a. How long have you been leaking watery, clear fluid?	☐ <24 hours (does not alter risk)<br>☐ 1-2 days (does not alter risk)<br>☐ 3-4 days (does not alter risk)<br>☐ >4 days (does not alter risk)
C6b. How much fluid are you leaking?	☐ A few drops (does not require a pad) (low risk)<br>☐ Moderate amount: intermittent with underwear wet (requires 1 pad per day) (moderate risk)<br>☐ Large amount: continuous trickle or gush (requires >1 pad per day) (high risk)
C7. Have you felt pressure in your pelvis or vagina since our last contact?	☐ Yes<br>☐ No
C7a. How long have you felt pressure?	☐ <24 hours (does not alter risk)<br>☐ 1-2 days (does not alter risk)<br>☐ 3-4 days (does not alter risk)<br>☐ >4 days (does not alter risk)
C7b. How would you describe the pressure that you feel?	☐ Very mild (low risk)<br>☐ Mild (low risk)<br>☐ Moderate (moderate risk)<br>☐ Strong (high risk)<br>☐ Severe (high risk)
C8. Have you experienced any other symptoms, not already addressed, that are concerning to you?	☐ Yes<br>☐ No
C8a. If yes, please specify:	
C9. Did you visit the OB triage or emergency department at the hospital this past week for pregnancy-related problems?	☐ Yes<br>☐ No
C9a. If yes, please provide the indication:	
C10. PTB assessment:	☐ <u>Low Risk</u>: No change in care recommended at this time.<br>☐ <u>Moderate risk</u>: These symptoms should be reported to your primary obstetrics provider at your next visit. If you have any concerns before your next visit, call your primary obstetrics provider.<br>☐ <u>High Risk</u>: These symptoms indicate a high risk for preterm birth. You should call your primary obstetrics provider immediately or go directly to the hospital where you plan to deliver for immediate evaluation.

[a] ACOG denotes American College of Obstetricians and Gynecologists, SMFM Society for Maternal-Fetal Medicine.
[b] ACOG/SMFM *Outpatient Assessment and Management for Pregnant Women with Suspected or Confirmed Novel Coronavirus (COVID-19)*. Available at https://s3.amazonaws.com/cdn.smfm.org/media/2263/COVID-19_Algorithm5.pdf; accessed January 22, 2024.

13. Table S3. Intent-to-treat analysis with multiple imputation for missing data.

MAR multiple imputation intent-to-treat (n=5018)	Ratio (95% CI)	*p* value	Ratio (95% CI)	*p* value
**Without covariates**	**Realized[a]**		**With death adjustment[b]**	
Neonatal morbidity and mortality[c]	0.80 (0.67-0.96)	0.019	0.81 (0.67-0.97)	0.020
Neonatal hospital length of stay[d]	0.96 (0.93-0.99)	0.018	0.92 (0.89-0.95)	<0.0001
NICU length of stay[e]	1.02 (0.97-1.08)	0.44	0.92 (0.87-0.96)	0.0011
NICU admission[f]	0.80 (0.67-0.96)	0.018	0.80 (0.67-0.96)	0.018
**With covariates**				
Neonatal morbidity and mortality[c]	0.80 (0.67-0.96)	0.018	0.80 (0.67-0.96)	0.019
Neonatal hospital length of stay[d]	0.96 (0.93-1.00)	0.025	0.92 (0.89-0.95)	<0.0001
NICU length of stay[e]	1.04 (0.98-1.09)	0.21	0.94 (0.89-0.99)	0.017
NICU admission[f]	0.80 (0.67-0.96)	0.018	0.80 (0.67-0.96)	0.018
**MNAR multiple imputation intent-to-treat (n= 5018)**	**Ratio (95% CI)**	***p* value**	**Ratio (95% CI)**	***p* value**
**Without covariates**				
Neonatal morbidity and mortality[c]	0.82 (0.68-0.98)	0.028	0.82 (0.68-0.98)	0.026
Neonatal hospital length of stay[d]	0.96 (0.93-0.99)	0.012	0.91 (0.88-0.94)	<0.0001
NICU length of stay[e]	1.02 (0.96-1.08)	0.51	0.91 (0.86-0.96)	0.0006
NICU admission[f]	0.80 (0.67-0.96)	0.017	0.81 (0.67-0.97)	0.019
**With covariates**				
Neonatal morbidity and mortality[c]	0.81 (0.68-0.98)	0.027	0.81 (0.68-0.97)	0.025
Neonatal hospital length of stay[d]	0.96 (0.93-0.99)	0.017	0.92 (0.89-0.95)	<0.0001
NICU length of stay[e]	1.03 (0.98-1.09)	0.24	0.93 (0.88-0.98)	0.008
NICU admission[f]	0.80 (0.67-0.96)	0.017	0.80 (0.67-0.97)	0.019

CI=confidence interval. MAR=missing at random. MNAR=missing not at random. NICU=neonatal intensive care unit.
Covariates were parity, pre-enrollment aspirin use, and COVID-19 positivity.
Ratios <1 indicate improved outcomes in the screen-guided care arm compared to the routine care arm.
[a] Cases of neonatal death used death as the length of stay.
[b] Cases of neonatal death assigned worst-case outcome (189 day stay).
[c] Odds ratio (OR); ordinal logistic regression.
[d] Incidence rate ratio (IRR); Poisson regression analysis.
[e] Incidence rate ratio (IRR); zero-inflated Poisson regression analysis.
[f] Logistic regression analysis.

14. Table S4. Baseline characteristics in the final modified intent-to-treat population.

Characteristic	Screen-guided care (N = 2143)	Routine care (N = 2325)	*p* value (N = 4468)
Maternal age – mean (SD) – yr	31 (6.0)	30 (6.0)	0.232
Race – no. (%)[a]			0.296
American Indian/Alaskan Native	7 (0.3%)	6 (0.3%)	
Asian	162 (7.6%)	158 (6.8%)	
Black/African American	296 (13.8%)	342(14.7%)	
Native Hawaiian/Pacific Islander	11 (0.5%)	12 (0.5%)	
White	1390 (64.9%)	1528 (65.7%)	
Multiracial	22 (1.0%)	38 (1.6%)	
Declined to answer	255 (11.9%)	241 (10.4%)	
Ethnic group – no. (%)[a]			0.984
Hispanic/Latina	368(17.2%)	402 (17.3%)	
Non-Hispanic/Non-Latina	1722 (80.4%)	1864 (80.2%)	
Declined to answer	53 (2.5%)	59 (2.5%)	
Insurance type – no. (%)			0.139
Medicaid	514 (24.0%)	590 (25.4%)	
Commercial	1591 (74.2%)	1678 (72.2%)	
Self-pay	38 (1.8%)	57 (2.5%)	
Gravidity – mean (SD)	2.31 (1.50)	2.35 (1.51)	0.442
Parity – no. (%)			0.960
Nulliparous	1039 (48.5%)	1129 (48.6%)	
Multiparous	1104 (51.5%)	1196 (51.4%)	
Prepregnancy BMI – mean (SD)	27.68 (7.31)	27.66 (7.03)	0.592
Preexisting diabetes (Type II) – no. (%)	60 (2.8%)	88 (3.8%)	0.066
Chronic hypertension – no. (%)	144 (6.7%)	152 (6.5%)	0.807
Pre-enrollment aspirin use (81 mg) – no. (%)	582 (27.2%)	639 (27.5%)	0.807

BMI=body mass index (kg/m^2). SD=standard deviation.
[a] Race and ethnicity were self-reported.

15. Table S5. Additional interim and final outcomes in the mITT and ITT populations.

Outcome	N	Screen-guided care	Routine care	Unadjusted (95% CI)	*p*	Adjusted (95% CI)[a]	*p*
**Interim mITT analysis**							
Neonatal morbidity	2944					0.74 (0.58-0.94)[b]	0.01
Neonatal hospital length of stay – days (mean)	442	6.97	17.02			0.49 (0.39-0.60)[c]	<0.001
**Final mITT analysis**							
Neonatal morbidity	4468			0.76 (0.63-0.91)[d]	0.004	0.75 (0.62-0.91)[d]	0.003
Neonatal hospital length of stay – days [mean (SD)]							
Entire study arm	4468	2.84 (6.48)	3.05 (7.90)	0.93 (0.90-0.97)[e]	<0.001	0.94 (0.90-0.97)[e]	<0.001
~10% quantile of longest stays	437	11.58 (18.37)	15.84 (27.85)	0.81 (0.67-0.97)[c]	0.025	0.82 (0.68-0.99)[c]	0.044
NICU admission [n/N (%)]	4468	211/2143 (9.8)	299/2325 (12.9)	0.74 (0.61-0.89)[f]	0.002	0.74 (0.61-0.89)[f]	0.002
NICU length of stay – days [mean (SD)]							
Entire study arm	4468	1.03 (6.67)	1.33 (8.09)	1.00 (0.95-1.06)[g]	>0.9	1.01 (0.96-1.07)[g]	0.7
~10% quantile of longest stays	436	10.81 (19.07)	14.67 (27.73)	0.81 (0.67-0.98)[c]	0.030	0.83 (0.69-1.01)[c]	0.067
Gestational age at birth -- weeks [mean (SD)]							
Entire study arm	4466	38.89 (1.47)	38.87 (1.70)	1.06 (1.00-1.12)[h]	0.043	1.05 (1.00-1.12)[h]	0.067
~10% quantile of earliest births	437	35.95 (2.01)	35.31 (2.67)	1.72 (1.40-2.11)[i]	<0.001	1.78 (1.45-2.19)[i]	<0.001
**ITT analysis**							
Neonatal hospital length of stay – days [mean (SD)]							
All ITT with data	4757	2.92 (6.74)	3.23 (9.61)	0.91 (0.88-0.93)[j]	<0.001	0.91 (0.88-0.94)[j]	<0.001
NICU length of stay – days [mean (SD)]							
All ITT with data	4759	1.10 (6.93)	1.50 (9.78)	0.90 (0.86, 0.95)[k]	<0.001	0.92 (0.88-0.97)[k]	0.003

Ratios <1 indicate improved outcomes in the screen-guided care arm compared to the routine care arm, except for gestational age at birth, where a ratio >1 denotes prolonged gestation.
CI=confidence interval. ITT=intention-to-treat. mITT=modified intention-to-treat. SD=standard deviation.

[a] Adjusted for parity, pre-enrollment aspirin use and COVID positivity for final analysis of mITT.
[b] Odds ratio; ordinal logistic regression. Data are based upon ordered categories (morbidity index score). Criteria for trail stoppage was based upon prespecified analyses that accounted for covariates of parity and pre-enrollment aspirin use only and met prespecified interim trial stoppage thresholds.
[c] Hazard ratio; Cox regression analysis in the approximate 10% quantile of longest stays in each arm. Data include the neonatal death adjustment.
[d] Data is based upon ranks. Odds ratio; ordinal logistic regression.
[e] Incidence rate ratio; Poisson regression analysis.
[f] Odds ratio; Logistic regression analysis.
[g] Incidence rate ratio; zero-inflated Poisson regression analysis.
[h] Hazard ratio; Cox regression analysis.
[i] Hazard ratio; Cox regression analysis in the approximate 10% of earliest births
[j] Incidence rate ratio; Poisson regression analysis. Data includes a prespecified neonatal death adjustment.
[k] Incidence rate ratio; zero-inflated Poisson regression analysis. Data includes a prespecified death adjustment.

16. Table S6. Final primary and secondary outcomes, including adjustment for covariates, in the modified intent-to-treat population.

Final mITT outcomes[a]	n	Adjusted ratio (95% CI)	*p* value
**Co-primary**			
**Neonatal hospital length of stay[b]**	437	0.82 (0.68-0.99)	0.044
Parity		0.98 (0.81-1.19)	0.90
Pre-enrollment aspirin use		0.97 (0.80-1.18)	0.80
COVID positivity		1.19 (0.80-1.76)	0.40
**Neonatal morbidity and mortality[c]**	4468	0.75 (0.62-0.91)	0.003
Parity		1.72 (1.42-2.09)	<0.001
Pre-enrollment aspirin use		1.84 (1.51-2.23)	<0.001
COVID positivity		1 (0.67-1.45)	1
**Co-secondary**			
**NICU length of stay[b]**	436	0.83 (0.69-1.01)	0.067
Parity		1.01 (0.83-1.22)	0.958
Pre-enrollment aspirin use		0.86 (0.71-1.05)	0.150
COVID positivity		1.15 (0.78-1.70)	0.482
**Gestational age at birth[d]**	437	1.78 (1.45-2.19)	<0.001
Parity		1.26 (1.04-1.52)	0.018
Pre-enrollment aspirin use		0.97 (0.80-1.18)	0.80
COVID positivity		0.88 (0.58-1.33)	0.60

CI=confidence interval. mITT=modified intention-to-treat. NICU=neonatal intensive care unit.
Ratio values <1 indicate improved outcomes in the screen-guided care arm compared to the routine care arm.
For gestational age at birth, a ratio >1 indicates prolonged gestation.
For covariates, the reference arm was multiparity, no pre-enrollment aspirin use, and no COVID positivity.
[a] Adjusted for parity, pre-enrollment aspirin use and COVID positivity.
[b] Hazard ratio; Cox regression analysis arm in the longest approximate 10% quantile of stays in each arm.
[c] Odds ratio; ordinal logistic regression.
[d] Hazard ratio; Cox regression analysis in the shortest approximate 10% quantile of gestation durations in each arm.

References

1. Iriye BK, Gregory KD, Saade GR, Grobman WA, Brown HL. Quality measures in high-risk pregnancies: Executive Summary of a Cooperative Workshop of the Society for Maternal-Fetal Medicine, National Institute of Child Health and Human Development, and the American College of Obstetricians and Gynecologists. Am J Obstet Gynecol 2017;217(4):B2–b25. (In eng). DOI: 10.1016/j.ajog.2017.07.018.
2. Bradford C, Severinsen R, Pugmire T, et al. Analytical validation of protein biomarkers for risk of spontaneous preterm birth. Clinical Mass Spectrometry 2017;3:25–38. DOI: 10.1016/j.clinms.2017.06.002.
3. Burchard J, Polpitiya AD, Fox AC, et al. Clinical Validation of a Proteomic Biomarker Threshold for Increased Risk of Spontaneous Preterm Birth and Associated Clinical Outcomes: A Replication Study. J Clin Med 2021;10(21):5088. DOI: 10.3390/jcm10215088.
4. U.S. Food and Drug Administration. E9(R1) Statistical Principles for Clinical Trials: Addendum: Estimands and Sensitivity Analysis in Clinical Trials: Guidance for Industry. (https://www.fda.gov/regulatory-information/search-fda-guidance-documents/e9r1-statistical-principles-clinical-trials-addendum-estimands-and-sensitivity-analysis-clinical). Accessed June 1, 2024.
5. NCSS Statistical Software. Group-Sequential Analysis for Two Hazard Rates. (https://www.ncss.com/wp-content/themes/ncss/pdf/Procedures/NCSS/Group-Sequential_Analysis_for_Two_Hazard_Rates.pdf). Accessed December 29, 2023.
6. Branch DW, VanBuren JM, Porter TF, et al. Prediction and Prevention of Preterm Birth: A Prospective, Randomized Intervention Trial. Am J Perinatol 2023;40(10):1071–1080. DOI: 10.1055/s-0041-1732339.
7. Hoffman MK, Kitto C, Zhang Z, et al. Neonatal Outcomes after Maternal Biomarker-Guided Preterm Birth Intervention: The AVERT PRETERM Trial. Diagnostics 2024;14(14):1462.
8. Hillemeier MM, Domino ME, Wells R, et al. Effects of maternity care coordination on pregnancy outcomes: propensity-weighted analyses. Matern Child Health J 2015;19(1):121–7. (In eng). DOI: 10.1007/s10995-014-1502-3.
9. Manuck TA, Henry E, Gibson J, et al. Pregnancy outcomes in a recurrent preterm birth prevention clinic. Am J Obstet Gynecol 2011;204(4):320 e1–6. (In eng). DOI: 10.1016/j.ajog.2011.01.011.
10. Meis PJ, Klebanoff M, Thom E, et al. Prevention of recurrent preterm delivery by 17 alpha-hydroxyprogesterone caproate. N Engl J Med 2003;348(24):2379–85. DOI: 10.1056/NEJMoa035140.
11. Newman RB, Sullivan SA, Menard MK, et al. South Carolina Partners for Preterm Birth Prevention: a regional perinatal initiative for the reduction of premature birth in a Medicaid population. Am J Obstet Gynecol 2008;199(4):393 e1–8. (In eng). DOI: 10.1016/j.ajog.2008.07.047.
12. Romero R, Conde-Agudelo A, Da Fonseca E, et al. Vaginal progesterone for preventing preterm birth and adverse perinatal outcomes in singleton gestations with a short cervix: a meta-analysis of individual patient data. Am J Obstet Gynecol 2018;218(2):161–180. (In eng). DOI: 10.1016/j.ajog.2017.11.576.
13. Romero R, Nicolaides KH, Conde-Agudelo A, et al. Vaginal progesterone decreases preterm birth </= 34 weeks of gestation in women with a singleton pregnancy and a short cervix: an updated meta-analysis including data from the OPPTIMUM study. Ultrasound Obstet Gynecol 2016;48(3):308–17. (In eng). DOI: 10.1002/uog.15953.
14. NCSS Statistical Software. NCSS PASS 2020. (https://www.ncss.com/software/pass/). Accessed October 15, 2024.
15. O'Brien PC, Fleming TR. A multiple testing procedure for clinical trials. Biometrics 1979;35(3):549–56. (In eng).
16. Holm S. A Simple Sequentially Rejective Multiple Test Procedure. Scand J Stat 1979;6(2):65–70.

17. Staffa SJ, Kohane DS, Zurakowski D. Quantile Regression and Its Applications: A Primer for Anesthesiologists. Anesth Analg 2019;128(4):820–830. DOI: 10.1213/ANE.0000000000004017.
18. Willke RJ, Zheng Z, Subedi P, Althin R, Mullins CD. From concepts, theory, and evidence of heterogeneity of treatment effects to methodological approaches: a primer. BMC Med Res Methodol 2012;12:185. DOI: 10.1186/1471-2288-12-185.
19. Merchant AT, Liu J, Reynolds MA, Beck JD, Zhang J. Quantile regression to estimate the survivor average causal effect of periodontal treatment effects on birthweight and gestational age. J Periodontol 2021;92(7):975–982. DOI: 10.1002/JPER.20-0376.
20. Berglund P, Heeringa S. Multiple Imputation of Missing Data Using SAS. Cary, NC, USA: SAS Institute Inc., 2014.
21. Arnold F, LaJoie A, Marrie T, et al. The pneumonia severity index predicts time to clinical stability in patients with community-acquired pneumonia. Int J Tuberc Lung Dis 2006;10(7):739–43. (https://www.ncbi.nlm.nih.gov/pubmed/16848334).
22. Bordon J, Peyrani P, Brock GN, et al. The presence of pneumococcal bacteremia does not influence clinical outcomes in patients with community-acquired pneumonia: results from the Community-Acquired Pneumonia Organization (CAPO) International Cohort study. Chest 2008;133(3):618–24. DOI: 10.1378/chest.07-1322.
23. Shindo Y, Sato S, Maruyama E, et al. Implication of clinical pathway care for community-acquired pneumonia in a community hospital: early switch from an intravenous beta-lactam plus a macrolide to an oral respiratory fluoroquinolone. Intern Med 2008;47(21):1865–74. DOI: 10.2169/internalmedicine.47.1343.
24. Schoenfeld D. Partial residuals for the proportional hazards regression model. Biometrika 1982;69(1):239–241. DOI: 10.1093/biomet/69.1.239.
25. Brown GW, Mood A. On median tests for linear hypotheses. Proceedings of the Second Berkeley Symposium on Mathematical Statistics and Probability. Berkeley, CA: University of California Press; 1951:159–166.
26. Brant R. Assessing proportionality in the proportional odds model for ordinal logistic regression. Biometrics 1990;46(4):1171–8.
